# TANK-binding kinase 1 protects against MASH progression via mitochondrial quality control

**DOI:** 10.1038/s12276-026-01672-9

**Published:** 2026-03-13

**Authors:** Sung-Min An, Jun Hee Jang, Jin Hyun Sung, Ji Won Myung, Yong Geun Jeon, Won Taek Lee, Jin Won Jeon, Kyung Min Yim, Jae-Ho Lee, Bichen Zhang, Jong Bae Seo, Seung Soon Im, Jae Bum Kim, Alan R. Saltiel, Jin Young Huh

**Affiliations:** 1 Center for Nano Materials, Department of Life Science, Sogang University, Seoul, Republic of Korea; 2 National Leading Researcher Initiatives Center for Adipocyte Structure and Function, Institute of Molecular Biology and Genetics, School of Biological Sciences, Seoul National University, Seoul, Republic of Korea; 3 Department of Physiology, College of Medicine and Institute of Medical Sciences, Gyeongsang National University, Jinju, Republic of Korea; 4 Department of Medicine, University of California, San Diego, San Diego, CA USA; 5 Department of Biomedicine, Health & Life Convergence Sciences, BK21 Four, Biomedical and Healthcare Research Institute, Mokpo National University, Jeonnam, Republic of Korea; 6 Department of Physiology, Keimyung University School of Medicine, Daegu, Republic of Korea; 7 Department of Pharmacology, University of California, San Diego, San Diego, CA USA

**Keywords:** Metabolic disorders, Organelles

## Abstract

Mitochondrial dysfunction is a critical driver of metabolic dysfunction-associated steatotic liver disease progression to steatohepatitis (MASH), yet the mechanisms governing mitochondrial quality control in hepatocytes remain poorly defined. Here we identify TANK-binding kinase 1 (TBK1) as an essential regulator of hepatic mitophagy and lysosomal activity. Using TBK1-deficient hepatocytes and liver-specific TBK1-knockout mice, we show that TBK1 loss leads to the accumulation of depolarized, reactive oxygen species-producing mitochondria due to impaired mitophagy flux, including defective lysosomal degradation. Mechanistically, TBK1 is required for p62 phosphorylation at Ser403 and partially modulates mTOR signaling to preserve lysosomal activity. Notably, both human samples and murine steatohepatitis models exhibited a substantial decline in TBK1 kinase activity. Therapeutic restoration of TBK1 expression via AAV8 delivery in MASH mouse model enhanced mitophagy, reduced mitochondrial burden and ameliorated liver fibrosis. Collectively, these findings establish TBK1 as a critical guardian of mitochondrial and lysosomal homeostasis in MASH.

## TBK1 Loss Impairs Mitochondrial Quality Control in Hepatocytes

Metabolic dysfunction-associated steatotic liver disease (MASLD) is a common liver condition linked to obesity. Researchers have found that a protein called TANK-binding kinase 1 (TBK1) plays a crucial role in managing the health of mitochondria in the liver. In this study, scientists used mice and human liver cells to explore how TBK1 affects liver health. They discovered that, when TBK1 is missing or not working properly, mitochondria become damaged and do not function well. This leads to increased stress and inflammation in liver cells. The researchers used various methods, including genetic modification and diet changes in mice, to study these effects. They found that TBK1 helps maintain mitochondrial quality by promoting a process called mitophagy, which removes damaged mitochondria. The study concludes that TBK1 is vital for preventing liver damage in MASLD by ensuring healthy mitochondria.

This summary was initially drafted using artificial intelligence, then revised and fact-checked by the author.

## Introduction

Metabolic dysfunction-associated steatotic liver disease (MASLD), previously known as nonalcoholic fatty liver disease, is a hepatic manifestation of metabolic syndrome and is closely correlated with obesity, one of the most substantial risk factors for the disease^1^. The global rise in obesity has led to an increased prevalence of MASLD, making it one of the most common chronic liver diseases worldwide^2^. The interplay between excessive lipid accumulation in hepatocytes and mitochondrial dysfunction is central to the pathogenesis of MASLD, particularly as the disease progresses toward metabolic dysfunction-associated steatohepatitis (MASH)^3^. Mitochondria are critical for fatty acid oxidation (FAO) and ATP production in the liver. However, in MASLD, the accumulation of lipids overwhelms mitochondrial capacity, leading to structural abnormalities such as fragmentation and swelling, as well as functional impairments such as reduced oxidative phosphorylation and increased reactive oxygen species (ROS) production^3–6^. These mitochondrial defects contribute notably to liver cell damage and the progression of MASLD to more severe stages, including MASH.

A key aspect of mitochondrial health is the maintenance of mitochondrial quality control mechanisms, which include mitochondrial biogenesis, fusion and fission, and mitophagy^7^. These processes are vital for maintaining mitochondrial function and preventing the accumulation of damaged organelles. Disruptions in these mechanisms are frequently observed in MASH, resulting in an imbalance between mitochondrial production and degradation, ultimately contributing to cellular dysfunction and disease progression^8^. Despite the recognized importance of mitochondrial dysfunction in MASH, the regulatory mechanisms underlying mitochondrial quality control remain poorly understood in the context of MASH.

Recent research has begun to elucidate the role of TANK-binding kinase 1 (TBK1), a serine/threonine kinase known for its involvement in multiple cellular processes, particularly autophagy^9^, innate immunity^10^ and energy metabolism^11–13^. Our previous study revealed that TBK1 plays a crucial role in hepatic lipid consumption via hepatic FAO^11^. The study demonstrated that liver (hepatocyte)-specific TBK1-knockout (LTKO) in mice led to reduced FAO via its scaffolding function, despite an increased number of mitochondria. This paradoxical increase in mitochondrial number suggested a complex regulatory role for TBK1 in mitochondrial quality control and underscored the importance of further investigating TBK1’s role in mitochondrial regulation, particularly in the context of MASH progression. We demonstrate here that TBK1 is a key regulator of hepatic mitophagy and mitochondrial quality control that is dysregulated in MASH. By investigating the function of TBK1 in MASH pathogenesis, we aim to provide new insights into the complex role of TBK1 in the progression of MASH.

## Materials and methods

### Animals and treatments

All mice were maintained on a C57BL/6J background. LTKO mice were generated by crossing TBK1 f/f (flox) mice^14^ with Albumin-Cre transgenic mice (Jackson Laboratory). Flox mice were bred with flox with Cre/+ mice to generate flox and flox ^Cre/+^ (LTKO) littermates. Only male mice were used for all experiments. To induce diet-induced obesity and metabolic dysfunction, 8-week-old flox and LTKO mice were fed a 60% high-fat diet (HFD; Research Diets, D12492) for 8 weeks. For fasting experiments, mice were subjected to an overnight fast (16 h) before sample collection. For the MASLD mouse model, *ob*/*ob* mice were fed either a normal chow diet (NCD) or a high-fat, high-fructose, high-cholesterol (Gubra-Amylin NASH, GAN) diet (Research Diets, D09100310) for 3–4 weeks, starting at 8 weeks of age, to mimic simple steatotic liver and MASH liver, respectively. Age-matched *ob*/+ mice were compared as lean controls. To overexpress human TBK1 in vivo, the cDNA sequence (NM_013254.3) was cloned into an AAV8 expression plasmid under the control of a CBh promoter (Vectorbuilder). For AAV infection experiments, AAV8-GFP or AAV8-human TBK1 (2 × 10¹¹ genome copies per mouse) was injected into 8-week-old *ob*/*ob* mice. Mice were allowed 3 weeks for TBK1 overexpression, after which they were placed on the GAN diet for an additional 4 weeks before tissue and blood collection. Mice were housed in a specific-pathogen-free facility under a 12-h light/dark cycle with ad libitum access to food and water, except during designated fasting periods. All experimental procedures were approved by the Institutional Animal Care and Use Committees at the University of California, San Diego, Seoul National University and Sogang University, and were conducted in accordance with institutional and national ethical guidelines.

### Generation of TBK1-KO HepG2 cells

RNA-guided targeting of human TBK1 in HepG2 cells was achieved through transfection of the Cas9 vector including guide RNA (gRNA). The gRNA was subcloned into Cas9 vector pSpCAS0(BB)-2A-Puro as described^15^. The 23-base-pair genomic targeting sequence of the human TBK1 gRNA was 5′-AAAATGTTTACAGCTTCCAG-3′. HepG2 cells were transfected with Cas9 vector containing gRNA using Lipofectamine 3000 (Invitrogen), and selected using 3 μg/ml of puromycin for 2 days. Selected cells were seeded by single cell in 96-well plates for generation of monoclonal cell line. The used monoclonal TBK1-deleted HepG2 cell was confirmed by detecting endogenous TBK1 protein by immunoblot.

### Mouse primary hepatocyte isolation

Mouse primary hepatocytes were isolated from 8–12-week-old NCD-fed flox or LTKO male mice or C57BL6/J male mice as described previously^11^. After 16 h of fasting, mice were infused through the inferior vena cava with 25 ml of perfusion buffer (138 mM NaCl, 5.4 mM KCl, 0.6 mM NaH_2_PO_4_·H_2_O, 0.8 mM Na_2_HPO_4_, 10 mM HEPES, 4.2 mM NaHCO_3_, 0.5 mM EGTA and 5 mM glucose, pH 7.4) for 3 min, followed by 25 ml of digestion buffer (40 µg/ml of Liberase TM (Roche), 138 mM NaCl, 5.4 mM KCl, 0.6 mM NaH_2_PO_4_·H_2_O, 0.8 mM Na_2_HPO_4_, 10 mM HEPES, 4.2 mM NaHCO_3_ and 5 mM CaCl_2_, pH 7.4) for 3 min using a peristaltic pump. Liver tissues were washed with 25 ml of perfusion buffer for 3 min after digestion. Excised livers were minced and centrifuged at 50*g* for 1 min. Dead cells were excluded by removing the floating cells after centrifugation at 100*g* for 10 min in 36% percoll solution (Cytiva, GE17089101). Cells were resuspended with William’s medium E without glutamine (Life Technologies, 12551-032) supplemented with 10% of fetal bovine serum (FBS), GlutaMax (Life Technologies, 35050-061), and 1% penicillin–streptomycin to make 3 × 10^5^ cells/ml. Then, 0.5 ml, 1 ml and 2 ml of cells were plated in 24-well, 12-well and 6-well collagen-coated plates. After 4-h incubation for cell attachment, the medium was replaced with fresh medium. All experiments with primary hepatocytes were performed within 48 h after isolation.

### Transmission electron microscopy for liver tissue

For mitochondrial morphology analysis for TBK1-deficient liver, 16-week-old flox and LTKO mice were used. The detailed procedure has been described previously^11^. For mitochondrial morphology analysis for AAV8-TBK1 overexpression, liver tissues were sliced at 2 mm × 2 mm × 2 mm cube and put in fixative overnight, postfixed in 1% osmium tetroxide in 0.1 M sodium cacodylate buffer for 1 h on ice, and stained en bloc with 0.5% uranyl acetate for overnight at 4 °C. The stained tissues were dehydrated in ethanol (30–100%) and embedded with Spurr’s resin. Ultrathin (50–60 nm) sections were poststained with uranyl acetate and lead stain. Samples were viewed using a JEOL JEM1010 (JEOL). For quantification, 48 images for AAV8-GFP group and 27 images AAV8-TBK1 overexpression group were analyzed using ImageJ.

### Human participants

The study protocol conformed to the ethical guidelines of the 1975 Declaration of Helsinki as reflected in a priori approval by the Ethics Committees of the First Affiliated Hospital of Keimyung University Dongsan Medical Center (no. DSMC 2022-03-011), and written informed consent was obtained from all participants. Human serum and liver tissues from patients with MASH at various stages were obtained from Keimyung University Dongsan Medical Center Biobank. Based on the patient dataset provided by human biobank at Dongsan Medical Center, the average and median ranges for normal patients (*n* = 36) and patients with MASH (*n* = 35) to represent the physical and biochemical parameters have been previously described^16^.

### qRT–PCR

For liver tissues, primary hepatocytes and HepG2 cell lines, RNA was isolated by using TRIzol reagent (15596018, Life Technologies). In total, 1–3 µg of RNA was used for reverse-transcription PCR to generate cDNA with ReverTra Ace qPCR RT kit (FSQ-101, Toyobo). The expression levels of mRNA were detected using the CFX96TM Real-Time System (Bio-Rad Laboratories). Quantitative reverse transcription polymerase chain reaction (qRT–PCR) was conducted using SYBR Green Master Mix (DQ384-40h, Biofact). *Cyclophilin* or *Tbp* was used as an endogenous control gene. Primer sequences are listed in Supplementary Table 1.

### Histology

Liver tissue was collected and fixed in 4% paraformaldehyde (Biosesang, P2031). Paraffin-embedding and sectioning for hematoxylin and eosin (H&E) staining were completed at the Woodang network in South Korea.

### FAO assay

FAO assays were performed as described previously^11^. Primary hepatocytes were incubated in serum-free William’s medium E for 16 h and then treated with FAO media (0.3% fatty-acid-free bovine serum albumin (BSA), 100 µM palmitate (Sigma-Aldrich, P9767), 0.4 µCi [1-^14^C] palmitate (PerkinElmer, NEC075H050UC), 1 mM carnitine (Sigma-Aldrich, 8.40092) in William’s medium E media in 24-well plates for 3 h at 37 °C. Then, 400 µl of the media was added to acidification vials, which had filter paper soaked with 40 μl of 1 M NaOH under the cap and 200 µl of 1 M perchloric acid in the tube. After 1 h, captured ^14^CO_2_ and acid-soluble metabolites (ASM) were used to measure radioactivity for FAO rates, and the CO_2_/ASM ratio was calculated to determine the complete oxidation rate. The cells on the plates were lysed with 0.1 M HCl to quantify protein concentrations.

### Measurement of mtDNA copy number

Mitochondrial DNA (mtDNA) copy number was analyzed as described in previous studies^17^. Same methods were used for HepG2 cell lines and liver tissues. In total, 20 mg of liver tissue was added to 600 μl of lysis buffer (0.2 mg/ml Proteinase K (Roche, 03115836001), 100 mM NaCl, 10 mM EDTA, 0.5% SDS and 20 mM Tris–HCl (pH 7.4)) and incubated overnight at 55 °C. Then, 100 µg/ml of RNase A (Roche Diagnostics, 10109169001) was added, and samples were incubated at 37 °C for 30 min. Samples were mixed with 250 μl of 7.5 M ammonium acetate and 600 μl of isopropyl alcohol and then centrifuged at 15,000*g* for 10 min at 4 °C. Pellets were washed with 70% ethanol, dried and solubilized in TE buffer. qRT–PCR was used to measure relative numbers of copies of mtDNA and nuclear DNA. mtDNA quantities were analyzed with mtDNA specific sequences and normalized with nuclear DNA sequences (GAPDH or hexokinase-2 (HK2)). Primer sequences are listed in Supplementary Table 2.

### Mitochondria fractionation for immunoblot analysis

Mitochondria was isolated as described in previous studies^18^. In brief, cells were minced in mitochondrial isolation buffer (MSHE; 70 mM sucrose, 210 mM mannitol, 5 mM HEPES and 1 mM EGTA, pH 7.2) by stroking five times with a Teflon glass homogenizer. The homogenate was centrifuged at 800*g* for 10 min, and the supernatant was centrifuged at 8,000*g* for 10 min to pellet the mitochondrial fraction. The pellet was completely resuspended with MSHE buffer and was centrifuged at 8,000*g* to wash the mitochondria pellet. The mitochondria pellet was resuspended with RIPA buffer to isolate protein samples. All steps for mitochondria isolation were performed on ice.

### Site-directed mutagenesis

P62 S403A and p62 S403E overexpression plasmids were generated by using CloneAmp Hifi DNA PCR premix (Takara, 639298) with 0.1 μg p62 wild-type (WT) plasmid. PCR products were treated with DpnI (NEB) to remove circular double-stranded templates from the reaction. After agarose gel purification, eluted DNA was ligated by using In-Fusion HD enzyme premix (Takara, 639690) and transformed into stellar competent cells (Clontech, 636763).

### Transfection

Mouse primary hepatocytes were transfected with small interfering RNA (siRNA; 5 pmol per well of a 24-well plate) against negative control or TBK1 by using Lipofectamine RNAiMAX (Life Technologies, 13778500). HepG2 cells were transfected with a combination of pcDNA-Flag-human TBK1 WT, pcDNA-Flag-human TBK1 kinase-dead mutant (K38A) plasmids, as described in each experiment, using Lipofectamine 3000 (Life Technologies). Primary hepatocytes were transfected with pcDNA-Flag-human TBK1 WT or pcDNA-Flag-human TBK1 K38A using Lipofectamine LTX (Life Technologies, A12621). Cells were collected after 48–72 h of transfection for further assays. For mitoKeima assay, mtKeima plasmid was overexpressed in TBK1 WT or TBK1 knockout (KO) via Lipofectamine 3000.

### Immunoblot analysis

Cells or liver tissues were lysed in RIPA buffer (25 mM Tris–HCl pH7.6, 150 mM NaCl, 1% NP-40, 1% sodium deoxycholate and 0.1% SDS) with 1 mM dithiothreitol and protease and phosphatase inhibitor cocktail (Thermo Fisher Scientific). After centrifuging the lysates at 14,000*g* for 15 min at 4 °C, the supernatant was used for protein quantification using BCA protein assay kits (Pierce, 23227). Primary antibodies were used at a 1:1,000 dilution and purchased from Cell Signaling: pS172 TBK1 (5483S), TBK1 (3031S), β-actin (4970), cleaved caspase 3 (9661), pY705 STAT3 (9131), STAT3 (9139), pT108/Y182 p38 (9211), p38 (9212), ATG12 (4180), ATG5 (12994), ATG7 (8558), pS403 p62 (39786), p62 (5114), TOM20 (42406), LC3 (12741), pS2481 mammalian target of rapamycin (mTOR; 2974), mTOR (2983), pT389 S6K (9205), S6K (9202), DYKDDDDK (Flag)-tag (14793), AMPK (2532), pT172 AMPK (2535) or Abcam: total OXPHOS(oxidative phosphorylation) antibody cocktail (ab110413), or Abclonal: LAMP1 (A2582), or BD Biosciences: RalA (610221).

### Immunoprecipitation

For Flag-tagged TBK1 overexpressed HepG2 cells, 500 μg of lysates were incubated with anti-Flag (Thermo Scientific Pierce, A36797) magnetic beads overnight at 4 °C on rotating shakers to pull down the protein complex with mTOR. The beads were rinsed three times for 10 min each in washing buffer (Tris-buffered saline containing 0.05% Tween-20 and 0.35 M NaCl) and eluted with sodium dodecyl sulfate (SDS) buffer. The eluates and input controls were then subjected to SDS–polyacrylamide gel electrophoresis and analyzed by immunoblotting.

### Fluorescence dye staining

In total, 500 nM of MitoTracker-DeepRed (Invitrogen, M46753) or 200 nM of MitoTracker-Green (Invitrogen, M46750) for mitochondria staining, 1 μM of LysoTracker Green (Invitrogen, L7526) for lysosome activity analysis, and 1 μM of BODIPY (Invitrogen, D3922) for neutral lipid accumulation analysis were incubated for 30 min before collecting cells. After staining, cells were analyzed via confocal microscope or flow cytometry. Then, 10 mg/ml of DQ-Red (Invitrogen, D12051) was incubated for 6 h before collecting cells.

### Mitochondrial quality measurement

1,1',3,3'-Tetraethyl-5,5',6,6'-tetrachloroimidacarbocyanine iodide (JC-1) dye (abcam, ab141387) or tetramethylrhodamine ethyl ester perchlorate (TMRE) dye (Invitrogen, T669) were used to analyze mitochondria membrane potential. In brief, cells were incubated with 5 μg/ml JC-1 dye at 37 °C for 1 h, and analyzed using FACS CantoII (BD Bioscience) or Cytoflex SRT (Beckman Coulter). For flow cytometry analysis, gates were set with unstained and carbonyl cyanide m-chlorophenyl hydrazone (CCCP)-treated cells and quantified emission filters appropriate for PE and FITC. The depolarized mitochondria ratio was calculated as the fluorescein isothiocyanate (FITC)/phycoerythrin (PE) ratio. For mitochondrial ROS measurement, 2 μM of mitoSOX (Invitrogen, M36009) was incubated for 30 min and then analyzed by FACS CantoII.

### Apoptosis analysis

Apoptosis was assessed using annexin-V conjugated with propidium iodide. WT and TBK1-deficient HepG2 cells were treated with 1 mM *N*-acetyl-L-cysteine (NAC; Sigma-Aldrich, A9165) for 6 h. After treatment, cells were washed twice with phosphate-buffered saline and stained with Annexin V apoptosis detection kit (BD Pharmingen, 556547).

### Quantification and statistical analysis

Statistical analyses were performed with Prism software version 10.4.0 (GraphPad Software). Comparisons of two groups were made by conducting Student’s *t*-tests. For more than two groups, we evaluated the data with one-way analysis of variance (ANOVA) or two-way ANOVA (Holm–Šidák’s multiple comparisons) test. Differences were considered statistically significant if *P* < 0.05 (* or #); *P* < 0.01 (** or ##); *P* < 0.001 (*** or ###); or *P* < 0.0001 (**** or ####). The statistical methods of each experiment are indicated in the figure legends.

## Results

### TBK1 is associated with maintenance of mitochondrial quality control

**Fig. 1:**
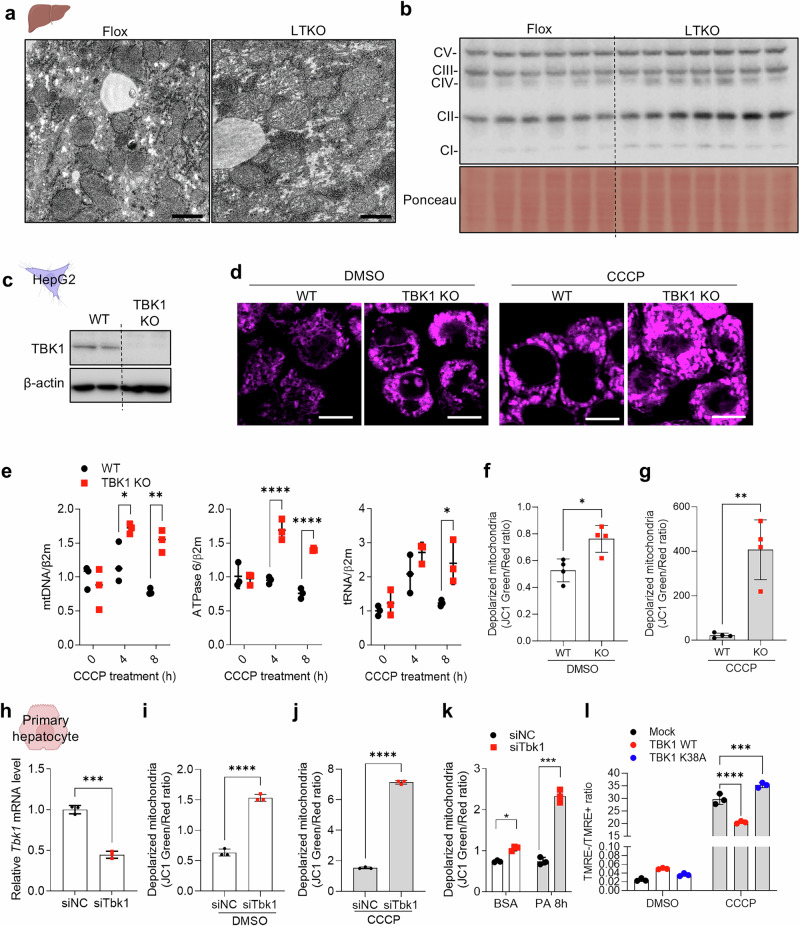
TBK1 is associated with maintenance of mitochondrial quality control. **a** Transmission electron microscopy (TEM) images of liver sections from HFD-fed flox and LTKO mice. Scale bar, 1 μm. **b** Immunoblot analysis of OXPHOS complex proteins in liver tissues from HFD-fed flox and LTKO mice (*n* = 6 or 7). **c**, Immunoblot of TBK1 protein levels in CRISPR–Cas9-mediated TBK1-KO HepG2 cells compared with WT HepG2 cells (*n* = 2). **d**, Representative images of MitoTracker Deep Red staining in WT or TBK1-KO HepG2 cells treated with dimethyl sulfoxide (DMSO) or 20 μM of CCCP for 4 h. **e**, mtDNA copy number in WT and TBK1 KO HepG2 cells following 20 μM of CCCP treatment, measured using mtDNA-specific primers (mtDNA, ATPase6 and tRNA). Nuclear DNA (β2-microglobulin) was used for normalization (*n* = 3). **f**, **g**, Quantification of mitochondrial depolarization under basal conditions (**f**) and CCCP treatment for 2 h (**g**) using JC-1 green/red fluorescence ratio in WT and TBK1 KO HepG2 cells (*n* = 4). **h**, *Tbk1* mRNA expression levels in mouse primary hepatocytes transfected with control siRNA (siNC) or *Tbk1*-targeting siRNA (siTbk1) (*n* = 3). **i**, **j**, Mitochondrial depolarization in siNC or siTbk1-transfected primary hepatocytes under basal (**i**) or CCCP-treated (**j**) conditions (*n* = 3). **k** Mitochondrial depolarization in *Tbk1*-knockdown hepatocytes treated with BSA or 200 μM palmitic acid for 8 h (*n* = 3). **l**, Mitochondrial depolarization measured by the ratio of TMRE-negative to TMRE-positive cells in primary hepatocytes overexpressing either WT TBK1 or kinase-dead TBK1 (K38A) (*n* = 3). *P < 0.05, **P < 0.01, ***P < 0.001, ****P < 0.0001. Data are presented as mean ± s.d. Statistical significance was determined by unpaired two-tailed Student’s *t*-test (**f**–**j**) or two-way ANOVA (**e**, **k** and **l**).

To investigate the role of TBK1 in hepatic function, we assessed the quantitative and qualitative changes in mitochondria induced by TBK1 deficiency in hepatocytes. We conducted a series of experiments using liver tissue from LTKO mice. Electron microscopy revealed that the mitochondria in the liver of LTKO mice appeared swollen, indicating morphological alterations associated with TBK1 deficiency (Fig. 1a), as shown in our previous study^11^. This alteration in mitochondria was further supported by a quantitative analysis of OXPHOS complex proteins, which showed a notable increase in their expression in LTKO liver tissue, consistent with previous reports^11^ (Fig. 1b).

To further explore the impact of TBK1 deficiency on mitochondrial quantity, we generated a TBK1-deficient HepG2 cell line using the CRISPR-Cas9 system. TBK1 KO was confirmed, and subsequent staining with MitoTracker revealed a obvious increase in mitochondrial mass in TBK1-deficient cells (Fig. 1c,d). Notably, when subjected to mitochondrial depolarization induced by CCCP, TBK1-deficient HepG2 cells exhibited an increase in mtDNA copy number, indicating a rise in mitochondrial quantity compared with control cells (Fig. 1e). However, mitochondrial biogenesis-related gene expression was not altered in TBK1 KO HepG2 cells (Supplementary Fig. 1a). These findings collectively suggest that TBK1 deficiency leads to an increase in mitochondrial quantity in hepatocytes without altering biogenesis.

In parallel with the observed increase in mitochondrial quantity, we assessed mitochondrial quality by measuring mitochondrial depolarization using JC1 dye staining. TBK1-deficient HepG2 cells showed a higher proportion of depolarized mitochondria under basal conditions, and this effect was more pronounced following CCCP treatment (Fig. 1f,g). To assess whether these findings extend to normal hepatocytes, we silenced TBK1 expression in mouse primary hepatocytes using siRNA. Consistent with observations in TBK1-deficient HepG2 cells, TBK1 knockdown led to increased mitochondrial depolarization both under basal conditions and following CCCP treatment (Fig. 1h–j) without substantial alterations in the expression of genes related to mitochondrial biogenesis (Supplementary Fig. 1b). To mimic the MASLD environment, we next treated TBK1-deficient cells with 200 µM palmitic acid, which led to a further increase in mitochondrial depolarization in the absence of CCCP (Fig. 1k). Moreover, overexpression of WT TBK1 ameliorated CCCP-induced mitochondrial depolarization, whereas expression of the K38A had no such effect (Fig. 1l). Collectively, these findings indicate that TBK1 deficiency results in the accumulation of dysfunctional mitochondria, underscoring its critical role in maintaining mitochondrial quality control in hepatocytes.

### TBK1-deficient hepatocytes exhibit increased susceptibility to mitochondrial stress

**Fig. 2:**
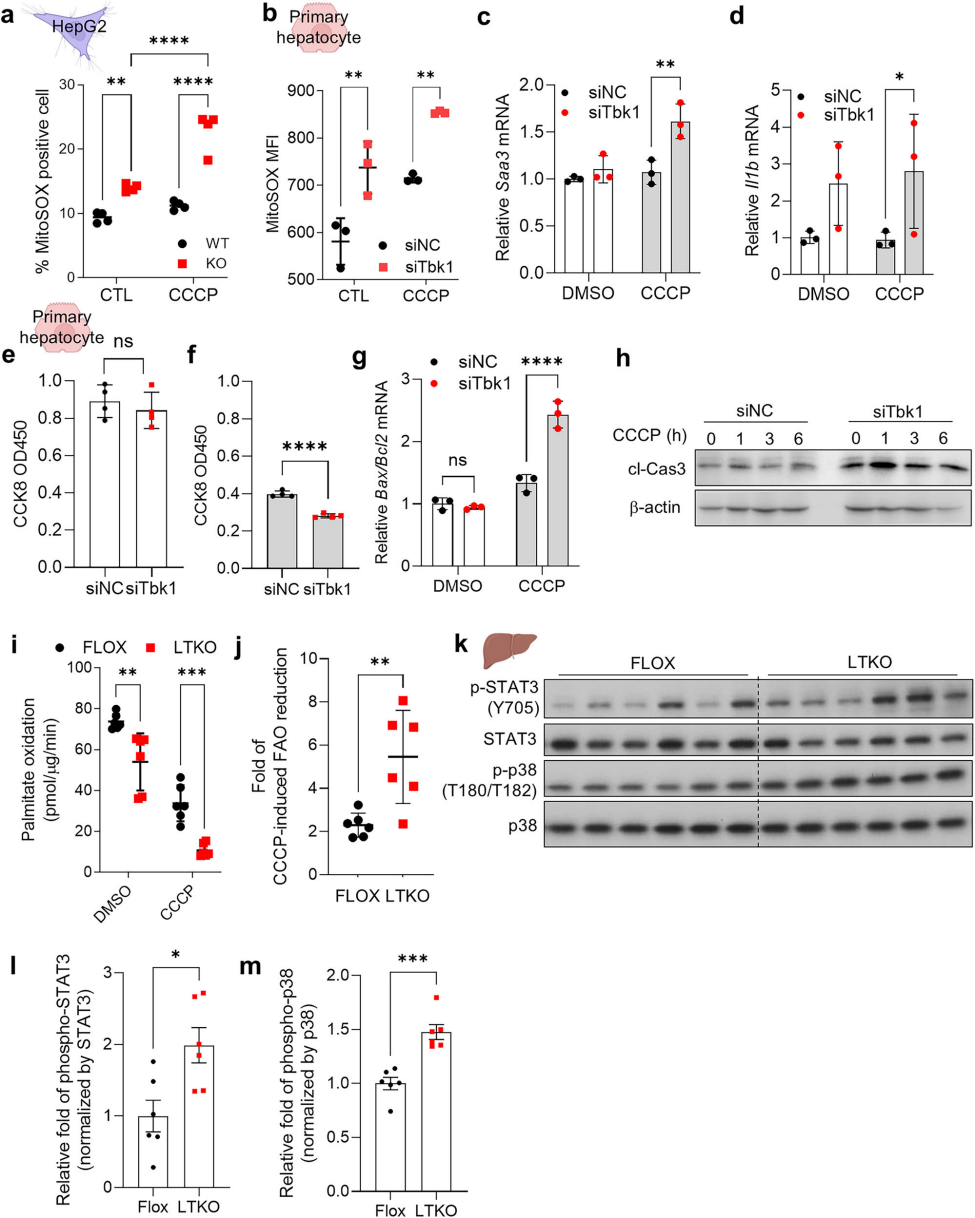
TBK1-deficient hepatocytes exhibit increased susceptibility to mitochondrial stress. **a** Mitochondrial ROS levels measured by MitoSOX staining in WT and TBK1 KO HepG2 cells (*n* = 4). **b**, Mitochondrial ROS levels in primary hepatocytes transfected with siNC or siTbk1 (*n* = 3). **c**, **d**, mRNA expression of proinflammatory genes *Saa3* (**c**) and *Il1b* (**d**) in siNC or siTbk1-transfected primary hepatocytes (*n* = 3). **e**,**f**, Cell viability measured by CCK-8 assay in primary hepatocytes under basal conditions (**e**) and following 10 μM of CCCP treatment (**f**) (*n* = 3). **g**, *Bax/Bcl2* mRNA expression ratio in primary hepatocytes under CCCP-induced stress (*n* = 3). **h**, Immunoblot of cleaved caspase-3 in primary hepatocytes treated with CCCP. **i**, Basal ^14^C-palmitate oxidation activity in primary hepatocytes from flox and LTKO mice (*n* = 6). **j**, Fold change in CCCP-induced suppression of palmitate oxidation activity in flox versus LTKO hepatocytes (*n* = 6). **k**, Immunoblot analysis of phosphorylated and total STAT3 and p38 in liver tissues from flox and LTKO mice (*n* = 6). **l**,**m**, Quantification of phospho-STAT3 (**l**) and phospho-p38 (**m**), normalized to their respective total protein levels (*n* = 6). *P < 0.05, **P < 0.01, ***P < 0.001, ****P < 0.0001. Data are presented as mean ± s.d. (**a**–**g**, **i** and **j**) or mean ± s.e.m. (**l** and **m**). Statistical significance was assessed by unpaired two-tailed Student’s *t*-test (**e**, **f**, **j**, **l** and **m**) or two-way ANOVA (**a**–**d**, **g** and **i**).

To investigate the impact of TBK1 deficiency on hepatocyte fitness through the accumulation of depolarized mitochondria, we first measured mitochondrial ROS. We observed a significant increase in mitochondrial ROS, as indicated by a mitoSOX signal^19^, in TBK1-deficient compared with WT HepG2 cells, indicating elevated mitochondrial ROS levels (Fig. 2a). Moreover, TBK1-deficient cells exhibited heightened sensitivity to mitochondrial ROS induction upon CCCP treatment, showing a more pronounced increase in ROS compared to WT cells. Similar patterns were observed in mouse primary hepatocytes with reduced TBK1 expression via siRNA, further confirming these findings (Fig. 2b). In addition, gene expression levels of inflammatory genes such as *Saa3* and *Il1b* were significantly enhanced in TBK1-deficient hepatocytes (Fig. 2c,d), possibly reflecting increased mitochondrial damage, although we note that TBK1 activity can inhibit noncanonical NFκB activation via phosphorylation and degradation of NIK in adipocyte^13^. In parallel, we assessed cell viability under these conditions using the Cell Counting Kit-8 (CCK-8) assay. We found that inhibition of TBK1 expression substantially increased CCCP-induced cell death (Fig. 2e,f). In addition, the ratio of *Bax*/*Bcl2* transcripts and cleaved caspase-3 levels were elevated in the *Tbk1* siRNA cells, suggesting that TBK1 may play a crucial role in maintaining cell viability under mitochondrial stress conditions (Fig. 2g,h). Also, mitochondrial ROS appeared to be one of the important drivers of this phenotype, as NAC treatment effectively reduced ROS levels and abolished the increase in Annexin-V-positive cells in TBK1-KO HepG2 cells (Supplementary Fig. 2). These results suggest that excessive mitochondrial ROS generation contributes substantially to the reduced cell viability observed under TBK1-deficient conditions. Furthermore, given the essential role of mitochondria in FAO, we measured FAO activity and found that basal FAO was reduced in TBK1 KO cells (Fig. 2i), consistent with previous reports^11^. Upon CCCP treatment, FAO was significantly decreased in TBK1 KO compared to WT cells, indicating a greater impairment of mitochondrial function in the absence of TBK1 (Fig. 2j). Consistent with upregulated mitochondrial ROS and stress responses in TBK1 KO hepatocytes in cell culture, LTKO mice also showed clearly increased phosphorylation of STAT3 and p38 in liver (Fig. 2k–m). These data suggest that TBK1 deficiency increases sensitivity to mitochondrial stress, leading to impaired mitochondrial function, such as reduced FAO, and heightened susceptibility to cell death as well as inflammation.

### TBK1 deficiency impairs the clearance of depolarized mitochondria in the liver

**Fig. 3:**
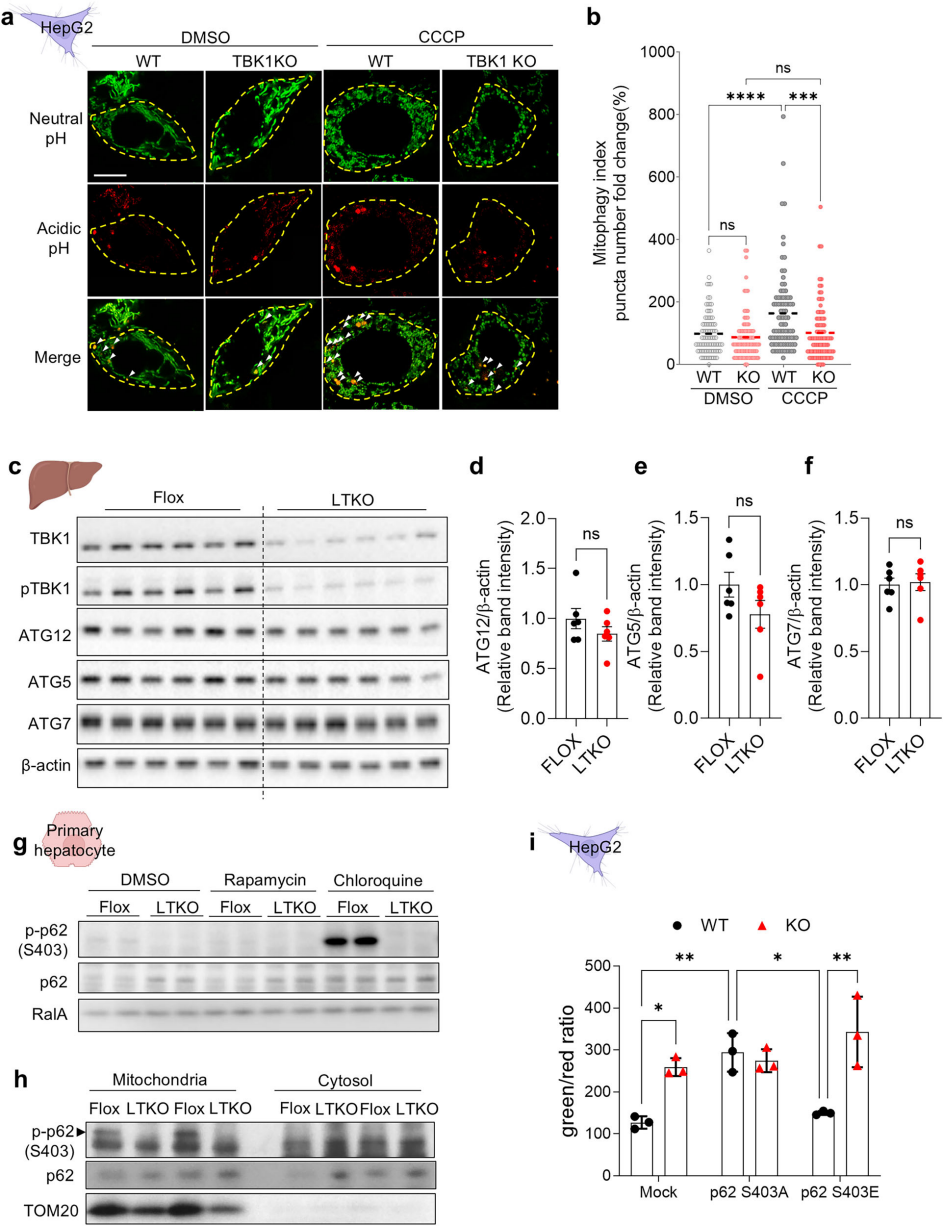
TBK1 deficiency impairs mitophagy, but the effect is not rescued by p62 phosphorylation. **a**, Representative images of mtKeima-based mitophagy assay in WT and TBK1 KO HepG2 cells under 20 μM of CCCP treatment for 4 h. **b**, Quantification of red puncta per cell representing mitolysosomes from **a**. **c**, Immunoblot analysis of autophagy-related proteins ATG12, ATG5 and ATG7 in liver tissues from flox and LTKO mice (*n* = 6). **d**–**f**, Quantification of ATG12 (**d**), ATG5 (**e**) and ATG7 (**f**) protein levels normalized to β-actin. **g**, Immunoblot analysis of phosphorylated p62 (Ser403) and total p62 in primary hepatocytes from flox and LTKO mice. **h**, Immunoblot analysis of phospho-p62 (Ser403) and total p62 in crude mitochondrial and cytosolic fractions of primary hepatocytes from flox and LTKO mice. **i**, Quantification of mitochondrial depolarization using JC-1 dye in TBK1 KO HepG2 cells overexpressing p62 S403A or p62 S403E mutants (*n* = 3). *P < 0.05, **P < 0.01, ***P < 0.001, ****P < 0.0001. Data are presented as mean ± s.d. (**b** and **i**) or mean ± s.e.m. (**d**–**f**). Statistical significance was determined using unpaired two-tailed Student’s *t*-test (**d**–**f**) or two-way ANOVA (**b** and **i**).

To investigate the mechanism underlying the increase in depolarized mitochondria observed in TBK1-deficient hepatocytes, we examined whether this was due to impaired clearance of depolarized mitochondria. A Mito-Keima assay was used to detect mitochondrial localization to lysosomes, which leads to degradation of damaged mitochondria. TBK1-KO HepG2 cells showed impaired mitochondrial localization into the lysosomal fraction upon CCCP treatment (Fig. 3a,b). Analysis of liver tissue from LTKO mice revealed no notable differences in protein expression levels of genes associated with general autophagy between genotypes (Fig. 3c–f). These findings imply that the impaired mitophagy in TBK1-deficient hepatocytes may not be attributed to altered abundance of core autophagy-related proteins such as ATG12, ATG5 and ATG7, suggesting a more specific disruption in mitophagy regulation rather than general autophagy machinery.

### TBK1 is necessary for p62 phosphorylation, but is not sufficient for mitophagy restoration in TBK1-deficient hepatocytes

To investigate the mechanism by which TBK1 regulates mitophagy in hepatocytes, we compared the recruitment of autophagy adaptor proteins, such as p62, which are known to be regulated by TBK1 in other cell types^20^. Primary hepatocytes were treated with rapamycin to induce autophagy. Phosphorylated p62 was barely detectable under these conditions. By contrast, treatment with chloroquine (CQ), which inhibits lysosomal degradation, led to the accumulation of phosphorylated p62 in WT cells, but not in TBK1-KO cells (Fig. 3g), indicating that TBK1 is essential for p62 Ser403 phosphorylation during autophagic flux. Consistent with impaired mitophagy, p62 protein levels were elevated in TBK1-deficient hepatocytes under basal conditions, while this difference was abolished upon CQ treatment, suggesting a defect in p62 turnover. Furthermore, phosphorylated p62 was preferentially enriched in the mitochondrial compared to the cytosolic fraction, a pattern that was lost in TBK1-KO cells (Fig. 3h), supporting a role for TBK1 in facilitating p62-mediated cargo recognition and mitochondrial targeting during mitophagy. To examine the potential role of p62 phosphorylation in mitophagy inhibition in TBK1-deficient cells, we overexpressed phospho-defective mutant p62 (S403A) and a phospho-mimetic mutant p62 (S403E) in TBK1-KO HepG2 cells and compared the level of mitochondrial depolarization. Notably, mitophagy was not restored by overexpression of the phospho-mimetic mutant p62 (Fig. 3i). This suggests that, while p62 S403E-mediated p62 activation enhances cargo delivery to lysosomes, its clearance might be impaired in the absence of TBK1. These findings indicate that p62 phosphorylation alone is insufficient to rescue mitophagy in TBK1-deficient cells.

### TBK1 deficiency shows defective lysosome activity

**Fig. 4:**
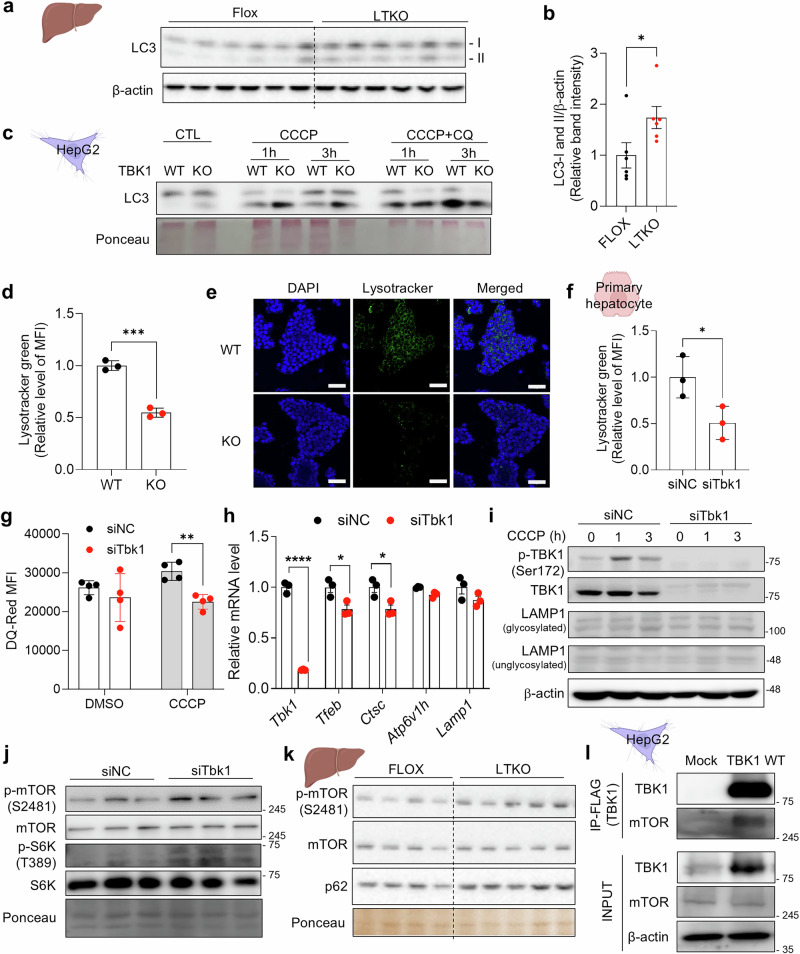
TBK1 deficiency shows defective lysosomal activity. **a**, Immunoblot analysis of LC3-I and LC3-II levels in liver tissues from HFD-fed flox and LTKO mice (*n* = 6). **b**, Quantification of LC3-I and LC3-II levels normalized to β-actin (*n* = 6). **c**, Immunoblot of LC3-I and LC3-II in WT and TBK1 KO HepG2 cells treated with CCCP or CCCP in combination with CQ for 6 h. **d**, Mean fluorescence intensity (MFI) of LysoTracker Green in WT and TBK1 KO HepG2 cells (*n* = 3). **e**, Representative confocal microscopy images of LysoTracker Green staining in WT and TBK1 KO HepG2 cells. Scale bar, 50 μm. **f**, MFI of LysoTracker Green in siNC- and siTbk1-transfected primary hepatocytes (*n* = 3). **g**, Lysosomal proteolytic activity assessed by DQ-Red BSA fluorescence in siNC- and siTbk1 primary hepatocytes (*n* = 4). **h**, Relative mRNA expression levels of lysosomal biogenesis-associated genes in siNC- and siTbk1-transfected primary hepatocytes (*n* = 3). **i**, Immunoblot of phosphorylated TBK1 (p-TBK1), total TBK1 and LAMP1 in siNC- and siTbk1-transfected primary hepatocytes upon CCCP treatment. **j**, Immunoblot analysis of phosphorylated mTOR and S6K levels in siNC- and siTbk1-transfected primary hepatocytes treated with 10 μM CCCP for 6 h. **k**, Immunoblot analysis of phosphorylated mTOR and mTOR levels in liver from NCD-fed flox versus LTKO mice (*n* = 4–5). **l**, Immunoprecipitation analysis for interaction between Flag-TBK1 and endogenous. *P < 0.05, **P < 0.01, ns, no significance (*P* > 0.05). Data are presented as mean ± s.d. (**d** and **f**–**i**) or mean ± s.e.m. (**b**). Statistical significance was assessed using unpaired two-tailed Student’s *t*-test (**b**, **d**, **f** and **h**) or two-way ANOVA (**g**).

Another notable phenotype observed in LTKO mice was a marked increase in both LC3-I and LC3-II levels in the liver (Fig. 4a,b), suggestive of altered autophagic flux. Further investigation revealed elevated LC3-II accumulation in the mitochondrial fraction of TBK1 KO HepG2 cells following CCCP treatment. Interestingly, this difference was abolished upon cotreatment with CQ, a lysosomal inhibitor, implicating defective lysosomal degradation as a key contributor to impaired mitophagy in TBK1-deficient cells (Fig. 4c). Moreover, combined CCCP and CQ treatment resulted in an overall reduction in total LC3 levels in the TBK1-KO group, suggesting that TBK1 deficiency not only disrupts lysosomal clearance but may also compromise the initiation of mitophagy. Collectively, these findings indicate that TBK1 is essential for effective lysosomal degradation of damaged mitochondria, and its loss leads to mitophagy impairment and the accumulation of dysfunctional mitochondria.

### Suppressed lysosomal activity is involved in impaired mitophagy in TBK1 deficiency

Given the observed inhibition of lysosomal degradation indicated by LC3 accumulation, we further investigated the changes in lysosomal activity caused by TBK1 deficiency. LysoTracker staining showed reduced intensity in TBK1-KO HepG2 cells, suggesting a decrease in basal lysosomal activity (Fig. 4d, e and Supplementary Fig. 3). This reduced lysosomal activity in TBK1 deficiency was consistently observed in TBK1-knockdown primary hepatocytes (Fig. 4f). Furthermore, DQ-Red BSA signal, which was used to assess lysosomal enzyme activity, decreased in siTbk1 hepatocytes following CCCP treatment (Fig. 4g).

To further assess the role of TBK1 in lysosomal biogenesis, we examined the mRNA levels of lysosome biogenesis-related gene expression in siTbk1 hepatocytes. Interestingly, knockdown of TBK1 led to a slight reduction of lysosomal markers, including transcription factor EB (*Tfeb*) and cathepsin C (*Ctsc*), while the levels of lysosomal-associated membrane protein 1 (LAMP1), a key lysosomal membrane protein, remained unaffected (Fig. 4h, i).

To explore further the mechanism of defective lysosomal activity in TBK1 deficiency, we analyzed the activation of the mTOR pathway, a known suppressor of lysosomal function^21^. In TBK1-deficient cells, basal phosphorylation of mTOR and of S6K was elevated, indicating a potential inhibitory role of TBK1 on mTOR activity (Fig. 4j). Consistently, liver tissues from LTKO mice also showed increased mTOR phosphorylation, in alignment with in vitro observations (Fig. 4k). In addition, given that previous reports have suggested functional crosstalk between TBK1 and mTOR signaling in other cell types^22–24^, we verified whether TBK1 directly interacts with mTOR in hepatocytes (Fig. 4l). Our data revealed that endogenous mTOR was robustly co-immunoprecipitated with TBK1, demonstrating a physical interaction between TBK1 and mTOR in hepatocytes. These findings collectively indicate that TBK1 contributes to the maintenance of lysosomal activity, potentially by suppressing mTOR signaling via direct interaction to support both lysosomal biogenesis and degradative capacity.

### TBK1 activity is reduced in MASH mouse model

**Fig. 5:**
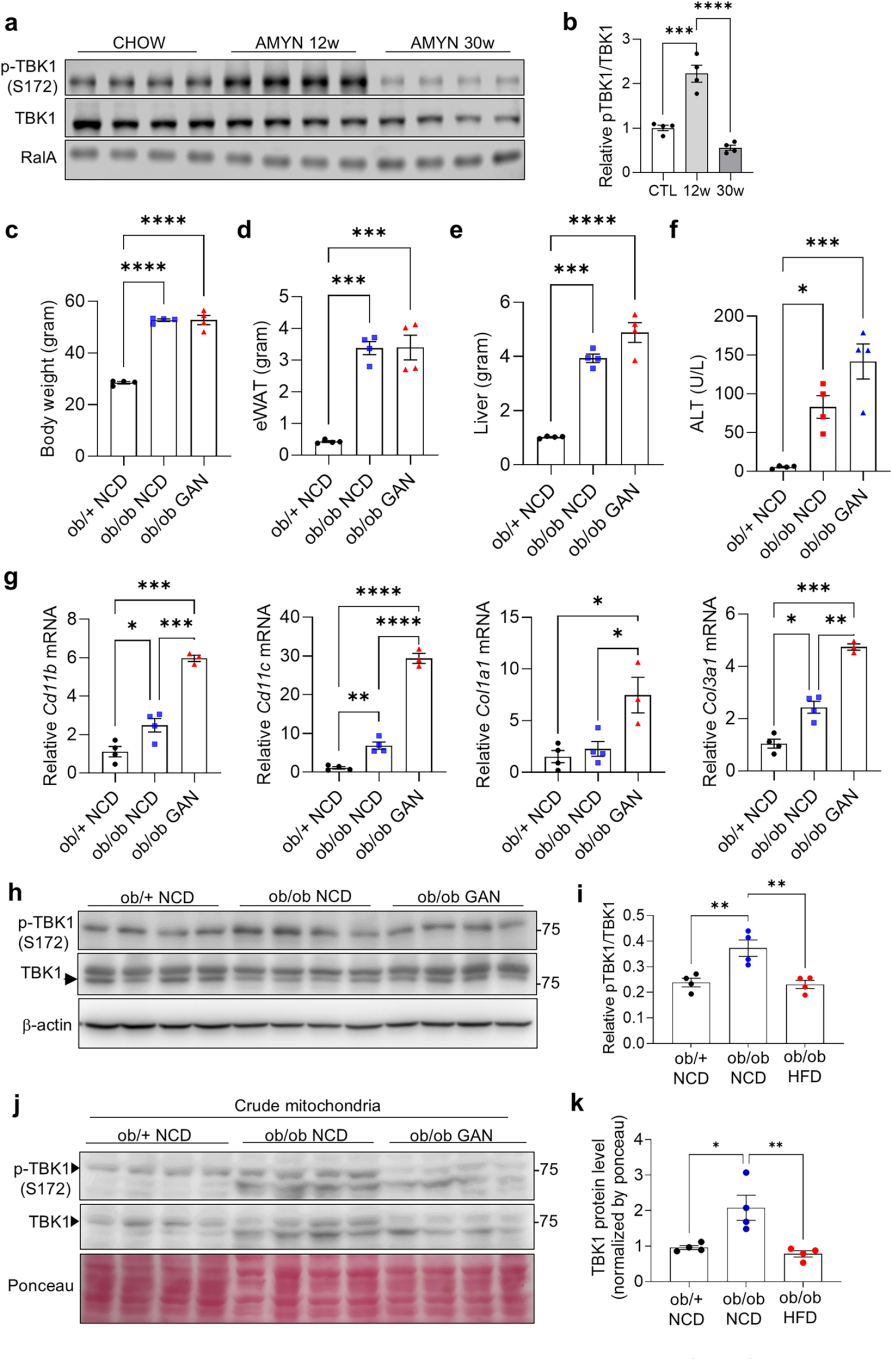
TBK1 kinase activity is reduced in MASH liver. **a**, Immunoblot analysis of phosphorylated TBK1 (p-TBK1) and total TBK1 in liver tissues from chow- or Amylin diet-fed mice (*n* = 4). **b**, Quantification of p-TBK1 levels normalized to total TBK1 in mitochondrial fractions (*n* = 4). **c**, Body weight of NCD-fed *ob*/+ (lean), NCD-fed *ob*/*ob* (steatosis) and GAN diet-fed *ob*/*ob* (MASH) mice (*n* = 4). **d**, eWAT weight across the same three groups (*n* = 4). **e**, Liver weight across the same three groups (*n* = 4). **f**, Serum ALT levels in the three groups (*n* = 4). **g**, mRNA expression levels of inflammatory and fibrosis-related genes in liver tissues from the three groups (*n* = 4). **h**, Immunoblot analysis of p-TBK1 and total TBK1 in liver lysates from NCD-fed *ob*/+, NCD-fed *ob*/*ob* and GAN-fed *ob*/*ob* mice (*n* = 4). **i**, Quantification of p-TBK1 levels normalized to total TBK1 (*n* = 4). **j**, Immunoblot of p-TBK1 and total TBK1 in crude mitochondrial fractions from NCD-fed *ob*/+ (lean), NCD-fed *ob*/*ob* (steatosis) and GAN diet-fed *ob*/*ob* (MASH) mice (*n* = 4). **k**, Quantification of mitochondrial TBK1 protein level (*n* = 4). *P < 0.05, **P < 0.01, ***P < 0.001, ****P < 0.0001. Data are presented as mean ± s.e.m. Statistical significance was determined using one-way ANOVA.

To investigate whether TBK1-mediated mitophagy might ameliorate MASH pathogenesis, we first assessed TBK1 expression and activity in liver tissues from MASLD mouse models. While TBK1 phosphorylation was markedly elevated after 12 weeks of Amylin diet, consistent with previous reports of increased activity after high-fat feeding^11^, long-term Amylin diet feeding (30 weeks), which induces MASH-like pathology, including fibrosis and hepatocellular death, resulted in a marked reduction in TBK1 phosphorylation, indicating impaired TBK1 activity (Fig. 5a,b). We generated a second MASLD mouse model, in which *ob*/*ob* mice were fed either a NCD or a GAN diet for 4 weeks to mimic steatosis or MASH stages, respectively. Compared with the lean control group (*ob*/+), both *ob*/*ob* NCD and *ob*/*ob* GAN diet groups had higher body weights, but no substantial difference was observed between the *ob*/*ob* NCD and *ob*/*ob* GAN groups (Fig. 5c). Similar patterns were observed for the mass of epididymal white adipose tissue (eWAT), liver weight and alanine aminotransferase (ALT) levels (Fig. 5d–f). Inflammatory markers, *Cd11b* and *Cd11c*, were significantly higher in the MASH stage, along with increased collagen transcript levels, indicating that the three groups successfully mimic healthy, steatosis and MASH stages (Fig. 5g).

The kinase activity of TBK1 was significantly reduced in *ob*/*ob* GAN diet-fed group compared with *ob*/*ob* NCD-fed group (Fig. 5h,i). In addition, public single-cell RNAseq data from the Liver Cell Atlas^25^ revealed decreased *Tbk1* mRNA expression in specific hepatocyte populations from MASLD mice (Supplementary Fig. 4).

The protein levels of phosphorylated TBK1 associated with mitochondria are reduced in *ob*/*ob* GAN diet compared with *ob*/*ob* NCD-fed mice (Fig. 5j,k). Consistent with impaired mitochondrial quality, OXPHOS complex proteins, which could reflect mitochondrial mass and respiratory capacity, were reduced in the *ob*/*ob* GAN group relative to other groups (Supplementary Fig. 5). Collectively, these results demonstrate that TBK1 kinase activity is progressively diminished during the transition from steatosis to MASH, particularly within hepatocyte mitochondria, highlighting a potential mechanistic link between TBK1 dysfunction and the exacerbation of MASH pathogenesis.

### TBK1 overexpression can ameliorate MASH development

**Fig. 6:**
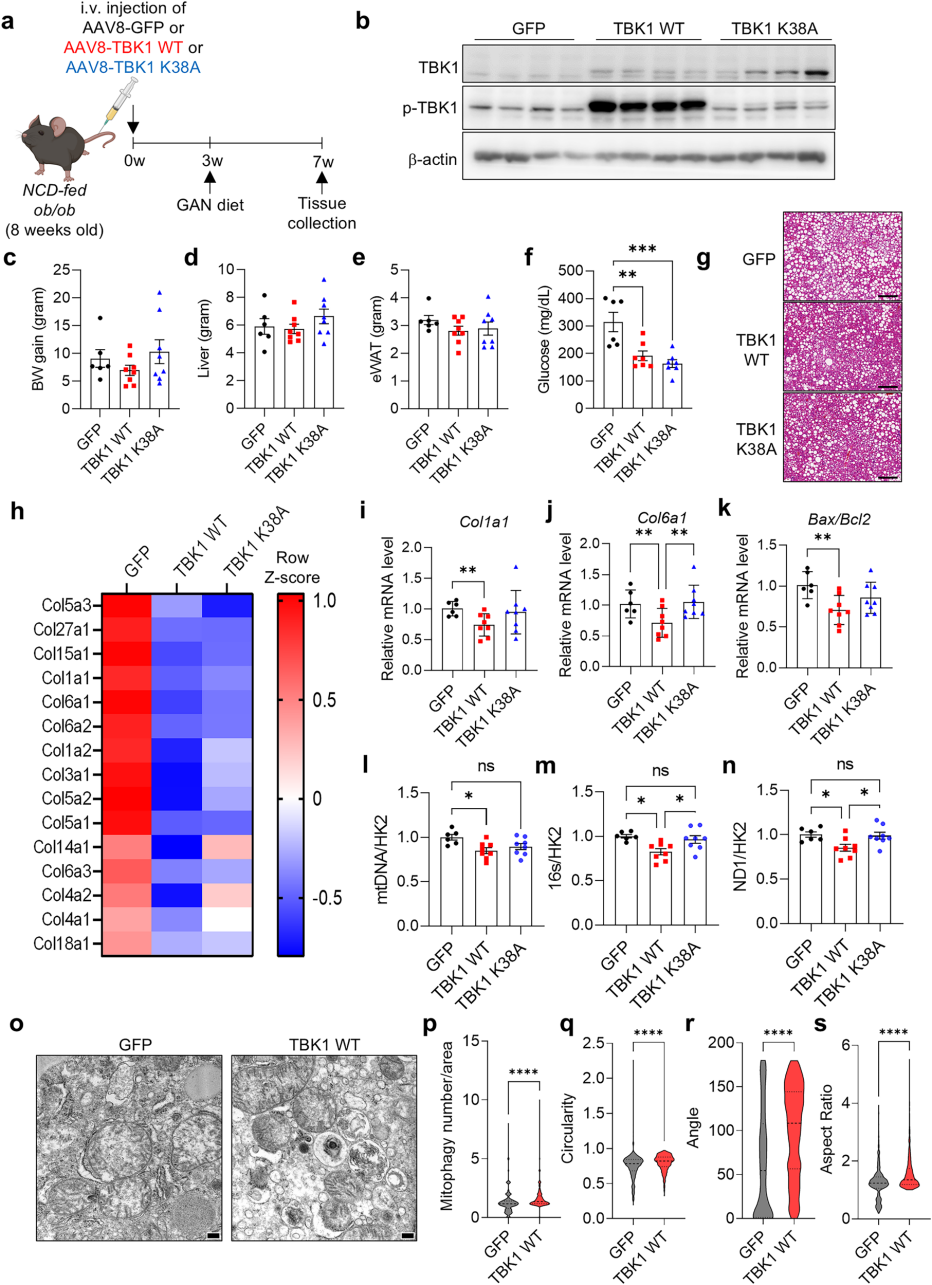
TBK1 overexpression ameliorates MASH progression in vivo. **a**, Experimental scheme for intravenous administration of AAV8-TBK1 WT or AAV-TBK1 K38A in the MASH mouse model. **b**, Immunoblot analysis of phosphorylated TBK1 (p-TBK1) and total TBK1 in liver tissues following AAV8-TBK1 WT or AAV-TBK1 K38A infection (*n* = 4). **c**–**e**, Body weight gain (**c**), liver weight (**d**) and eWAT weight (**e**) (*n* = 6). **f**, Fasting blood glucose levels (*n* = 6). **g**, Representative H&E-stained liver histological images from each group. Scale bar, 200 μm. **h**, Relative mRNA expression of fibrosis-related collagen genes in liver tissues (*n* = 3). **i**–**k**, qPCR analysis of *Col1a1* (**i**), *Col6a1* (**j**) and *Bax/Bcl2* ratio (**k**) in liver tissues (*n* = 6). **l**–**n**, mtDNA copy number normalized to HK2 in liver tissues (*n* = 6). **o**, Transmission electron microscopy (TEM) images of liver tissues. Scale bar, 200 nm. **p**–**s**, Quantification of mitochondrial morphology by TEM: mitophagy-associated vesicular mitochondria per area (**p**), mitochondrial circularity (**q**), angle (**r**) and aspect ratio (**s**) (*n* = 6). *P < 0.05, **P < 0.01, ***P < 0.001, ****P < 0.0001, ns, no significance (*P* > 0.05). Data are presented as mean ± s.e.m. Statistical significance was determined using one-way ANOVA (**c**–**f** and **i**–**n**) or unpaired two-tailed Student’s *t*-test (**p**–**s**).

To determine whether MASH progression can be ameliorated by overexpressing TBK1, AAV8-TBK1 WT or AAV-TBK1 K38A (kinase dead mutant) was administered intravenously to *ob*/*ob* mice, followed by a MASH-inducing GAN diet for 4 weeks (Fig. 6a). Immunoblot analysis confirmed TBK1 overexpression (Fig. 6b). Both WT and K38A overexpression did not affect body weight, liver weight or eWAT weight (Fig. 6c–e). However, blood glucose levels significantly decreased in both WT and K38A overexpression groups (Fig. 6f). Consistent with previously described kinase-independent actions of TBK1 on FAO^11^, H&E staining revealed reduced hepatic lipid accumulation in mice overexpressing either WT or K38A (Fig. 6g).

RNA sequencing analysis of liver tissues revealed that TBK1 WT overexpression markedly downregulated extracellular matrix-related transcripts, particularly fibrosis-associated collagens, whereas the K38A mutant showed only modest effects (Fig. 6h). Kyoto Encyclopedia of Genes and Genomes (KEGG) pathway enrichment further demonstrated that TBK1 WT increased lysosome-associated gene expression (Supplementary Fig. 6a). Although K38A overexpression produced partially overlapping effects, it also displayed a distinct gene signature characterized by increased fatty acid metabolism pathways (Supplementary Fig. 6b). Direct comparison of WT versus K38A overexpression confirmed that TBK1 WT uniquely upregulated lysosomal and oxidative phosphorylation gene programs, implying a more direct role for TBK1 kinase activity in regulating mitochondrial and lysosomal function (Supplementary Fig. 6c). qRT–PCR further confirmed reduced expression of fibrosis-related genes (*Col1a1* and *Col6a1*) and *Bax*/*Bcl2* gene expression ratio, indicative of cell death markers, in WT but not K38A (Fig. 6i–k). These findings indicate that TBK1 kinase activity is crucial for the activation of lysosomal pathways, as well as for the repression of extracellular matrix-, fibrosis- and cell death-associated genes. To assess whether TBK1 overexpression enhances mitophagy, we measured mtDNA copy number. It was considerably reduced in TBK1 WT-overexpressing livers but remained unchanged in mice expressing the K38A (Fig. 6l–n), supporting an important role of TBK1 kinase activity in promoting mitochondrial clearance. Collectively, these results suggest that, although some metabolic improvements—such as reduced steatosis and lower glucose levels—are shared by WT and K38A, TBK1 kinase activity exerts a more direct role in activating lysosomal and mitochondrial quality control pathways and in suppressing fibrosis-associated gene expression during MASH progression.

Electron microscopy analysis showed an increase in autophagosome-enclosed mitochondria, indicative of ongoing mitophagy, which was quantified and confirmed to increase with TBK1 WT (Fig. 6o,p). Other mitochondrial morphology features—such as reduced circularity, increased angle and higher aspect ratio—reflect improved mitochondrial quality in the TBK1 WT group (Fig. 6q–s). These findings suggest that TBK1 WT overexpression mitigates MASH pathogenesis by promoting mitophagy and reducing fibrosis.

In addition, to strengthen the evidence that TBK1 restoration ameliorates MASH-associated phenotypes, we overexpressed TBK1 in primary hepatocytes and treated the cells with CCCP alone or cotreated with CCCP and CQ to inhibit lysosomal activity. As shown in Supplementary Fig. 7a, CCCP treatment strongly induced inflammatory genes whereas TBK1 overexpression attenuated their induction. Notably, cotreatment with CCCP and CQ partially abolished the TBK1-mediated suppression of inflammatory gene expression, suggesting that lysosomal activity contributes to TBK1-dependent regulation of inflammation. In parallel, lipid accumulation was quantified using BODIPY staining (Supplementary Fig. 7b). TBK1 overexpression substantially reduced basal lipid content. Lipid levels remained similarly reduced by CQ treatment. However, CCCP-induced lipid accumulation was not rescued by TBK1 overexpression, and cotreatment with CCCP and CQ even showed a tendency toward increased lipid content. These findings suggest that TBK1-mediated suppression of hepatic steatosis may occur through lysosome-independent mechanisms. Altogether, these results support a protective role for TBK1 in mitigating hepatocyte inflammation and lipid accumulation, consistent with its in vivo effects on MASH-related steatosis and fibrosis.

### TBK1 activity shows a negative correlation with severity of MASLD in patients

**Fig. 7:**
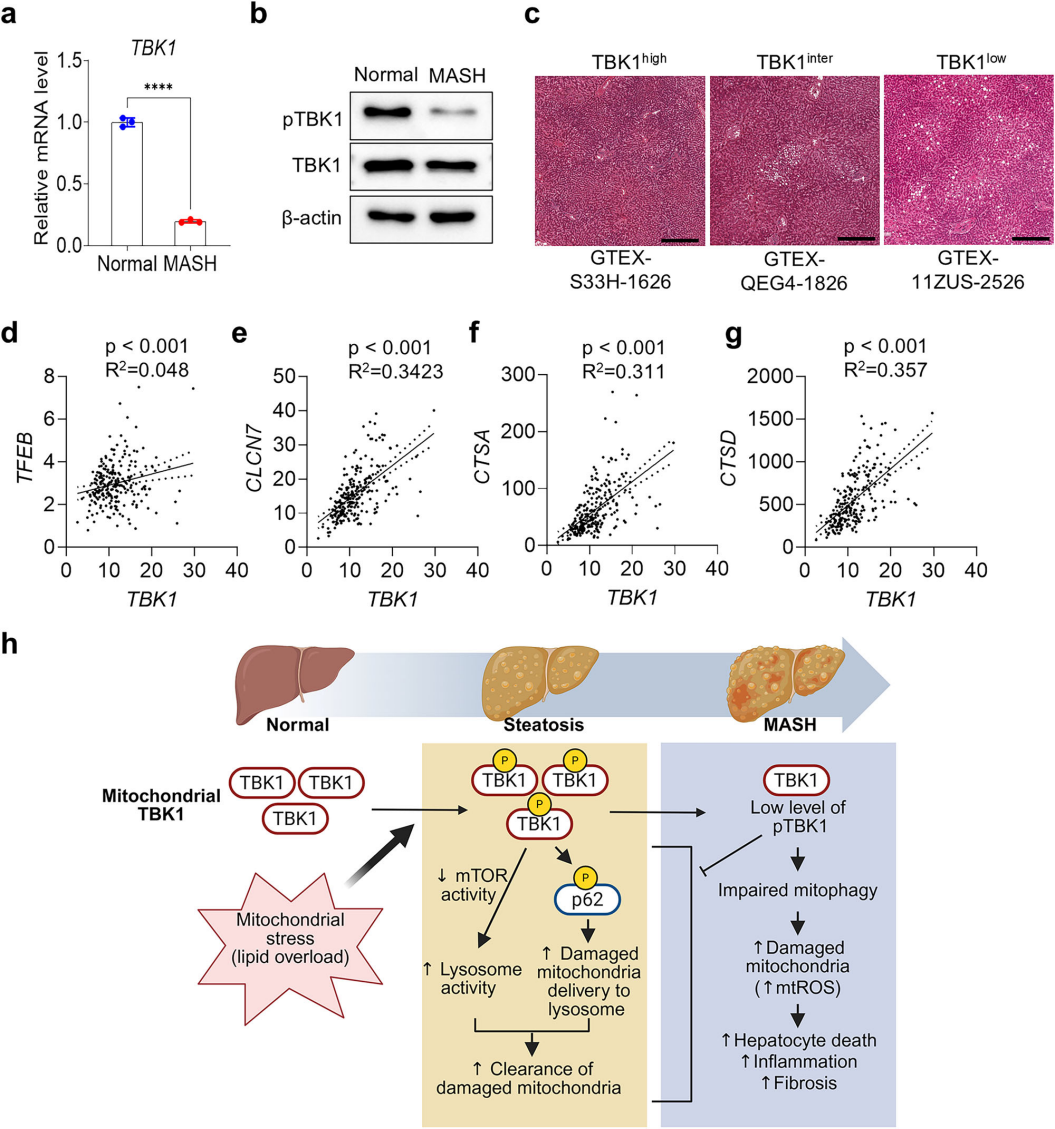
TBK1 activity shows a negative correlation with MASLD in patients. **a**, Relative mRNA expression levels of TBK1 in liver tissues from normal participants and patients with MASH (*n* = 3 per group). **b**, Immunoblot analysis of phosphorylated TBK1 (p-TBK1) and total TBK1 in liver lysates from normal and MASH patient samples. **c**, Representative H&E-stained liver histological sections stratified by TBK1 expression levels (high, medium and low). Scale bars, 400 µm. **d**–**g**, Simple linear regression analysis showing the correlation between *TBK1* mRNA levels and lysosomal biogenesis–associated genes, including *TFEB* (**d**), *CLCN7* (**e**), *CTSA* (**f**) and *CTSD* (**g**) across human liver samples. The data used for the analyses were obtained from: dbGaP accession number phs000424.v8.p2. **h**, Proposed model. Upon mitochondrial stress, phosphorylated TBK1 enhances mitochondrial quality control by promoting p62 phosphorylation and lysosomal activity. In MASH liver, however, both the activation and mitochondrial localization of TBK1 are diminished, resulting in impaired mitophagy and the accumulation of damaged mitochondria. This dysfunction exacerbates mitochondrial stress, inflammation and fibrosis, thereby accelerating MASH progression. ****P < 0.0001. Data are presented as mean ± s.e.m. Statistical significance was determined by unpaired two-tailed Student’s *t*-test (**a**) or linear regression analysis (**d**–**g**).

Analysis of human MASH samples showed reduced levels of both total TBK1 and phosphorylated TBK1 (Fig. 7a,b). Increased lipid accumulation in liver was correlated with lower TBK1 expression based on GTEx histology data (Fig. 7c). Furthermore, the mRNA expression level of lysosome-related genes, such as *TFEB*, *CLCN7*, *CTSA* and *CTSD*, showed a positive correlation with *TBK1* expression in human liver (Fig. 7d–g). These findings highlight the potential role of TBK1 in the regulation of mitophagy and the progression of MASH.

## Discussion

We report here a role for the protein kinase TBK1 in the control of mitochondrial morphology and function. TBK1-deficient hepatocytes and liver tissues from LTKO mice showed increased mitochondrial number but compromised quality, as indicated by elevated mitochondrial depolarization and ROS levels. Impaired lysosomal activity and reduced mitophagy flux were identified as underlying mechanisms contributing to the accumulation of dysfunctional mitochondria in TBK1-deficient cells. Importantly, TBK1 overexpression improved mitochondrial quality and reduced markers of fibrosis in MASH mouse models, with enhanced mitophagy and decreased level of fibrosis-related gene expression, emphasizing the essential role of TBK1 kinase activity in restoring cellular homeostasis in the liver. Our findings also highlight a decrease in TBK1 activity in MASH, observed in both mouse models and human samples, supporting the pathological relevance of TBK1 dysregulation in late stages of MASLD progression (Fig. 7h).

Mitophagy, the selective autophagic degradation of damaged mitochondria, is a vital process for maintaining mitochondrial quality and cellular homeostasis in hepatocytes^26,27^. Previous studies have shown that TBK1 regulates mitophagy by phosphorylating autophagy adaptor proteins such as OPTN, NDP52 and p62, enabling the recruitment of damaged mitochondria to the autophagy machinery^20,28,29^. However, many studies were conducted in nonhepatic cells or artificial settings, such as PENTA KO systems, which lack multiple cargo receptors including OPTN, CDP52, TAX1BP1, p62 and NBR1^9^. Our study builds on these findings by showing that TBK1 is essential for mitophagy in normal hepatocytes, highlighting its physiological relevance in hepatic mitochondrial quality control. Also, we observed p62 Ser403 phosphorylation as a TBK1-dependent mechanism that enhances cargo delivery to lysosomes during hepatic mitophagy. Interestingly, because p62 Ser403 phosphorylation was insufficient to rescue mitophagy in TBK1-deficient cells, these findings imply that, while p62 S403 phosphorylation is crucial for targeting damaged mitochondria to the autophagic machinery, efficient mitochondrial clearance ultimately requires intact lysosomal function.

Notably, our results highlight the role of TBK1 as a regulator of lysosomal activity in hepatocytes. Lysosomal dysfunction, indicated by reduced LysoTracker staining intensity and DQ-Red BSA fluorescence, was observed in TBK1-deficient cells. Although lysosomal quantity (for example, LAMP1 levels) was not affected, TBK1 deficiency impaired lysosomal degradation capacity, contributing to the accumulation of damaged mitochondria. This impaired lysosomal activity may provide a mechanistic explanation for previous observations of increased transfection efficiency in TBK1-deficient cells^30^. Given that agents such as CQ enhance transgene expression by preventing lysosomal DNA degradation, the loss of TBK1 may similarly suppress lysosomal function, thereby stabilizing transfected plasmids and facilitating gene expression. Furthermore, our data indicate that impaired lysosomal activity in TBK1-deficient hepatocytes is closely linked to increased inflammation. TNFα reduced lysosomal activity, and blocking lysosomal degradation during mitochondrial stress further amplified inflammatory gene expression (Supplementary Fig. 8). These findings suggest a mutual reinforcement between lysosomal dysfunction and inflammation, providing a mechanistic basis for the heightened inflammatory signaling observed in TBK1-deficient cells.

TBK1-deficient cells also showed hyperactivation of mTOR signaling, which is known to suppress lysosomal biogenesis and activity^31^. These data suggest that the regulatory role of TBK1 in lysosomal activity may be partially mediated through mTOR modulation. The regulatory relationship between TBK1 and mTOR signaling is complex and appears highly context dependent, varying with cell type, nutrient status and disease stage^22,23,32,33^. Several studies have reported that TBK1 negatively regulates mTORC1 activity through phosphorylation of Raptor at Ser877, particularly under chronic innate immune activation, such as in Trex1-deficient or prostate cancer models, where TBK1 suppresses mTOR-driven anabolic programs and promotes cellular quiescence or dormancy^22^. Conversely, recent findings suggest that lysosome-associated TBK1 may facilitate mTORC1 activation by relieving Rab7-mediated suppression under amino acid-rich conditions^33^. In our study, TBK1-deficient hepatocytes showed an increase in mTOR signaling, coupled with a marked reduction in lysosomal activity and impaired mitophagy flux. Notably, we found that TBK1 physically interacts with mTOR in hepatocytes. This interaction demonstrates that TBK1 can directly coordinate mTOR signaling, suggesting hepatic TBK1 may play a supportive role in lysosomal maintenance, thereby restraining mTORC1 activity under basal or stressed conditions by sustaining lysosomal degradation capacity.

Our findings suggest a dynamic, stage-dependent regulation of TBK1 kinase activity during the progression of MASLD and MASH. Phosphorylation of TBK1 (Ser172), a marker for its kinase activity, is increased during the early stages of hepatic steatosis—probably in response to inflammatory cues. This observation aligns with prior reports indicating TBK1 activation under inflammatory stress^11^. We propose that, during the initial phase of MASLD, TBK1 is phosphorylated as an adaptive response, promoting mitophagy to remove damaged mitochondria and maintain mitochondrial homeostasis. However, as the disease advances toward MASH, TBK1 kinase activity becomes markedly diminished despite relatively preserved total protein levels in mice, with reduced TBK1 expression in liver tissue of human MASH patients.

Identifying upstream mechanisms underlying impaired TBK1 activity in the latter stages of MASH remains an important avenue for future research. AMPK is a potential candidate, as it has been shown to phosphorylate TBK1 at Ser511 in response to viral infection, facilitating downstream IRF3 recruitment and innate immune activation^34^. In support of this, we have also observed that AMPK can indirectly activate TBK1 via ULK1 in adipocytes, suggesting that this axis may serve as a general mechanism linking metabolic stress to TBK1 activation^13^. Given that AMPK activity is diminished in MASH livers^35^, defective AMPK–TBK1 signaling could contribute to impaired mitophagy and mitochondrial dysfunction. In addition, the reduction in TBK1 activity may be, at least in part, a secondary effect of hepatocyte injury or loss in advanced stages of disease. Distinguishing primary regulatory defects from downstream consequences of liver damage will be critical for fully understanding TBK1’s role in MASH progression. Because the AMPK–TBK1 axis differs across cell types, understanding how this regulatory pathway operates in hepatocytes is important.

Notably, the functional role of TBK1 appears to differ between liver and adipose tissue. In both tissues, TBK1 deletion upregulates inflammation^11,13^, indicating that its anti-inflammatory function is shared across metabolic tissues. However, the metabolic consequences of TBK1 loss diverged substantially. In adipocytes, TBK1 deficiency resulted in reduced adiposity and increased energy expenditure driven by robust activation of AMPK^13^. By contrast, hepatocyte-specific TBK1 KO mice did not show AMPK hyperactivation under NCD-fed condition (Supplementary Fig. 9). Instead, they displayed impaired FAO and increased triglyceride accumulation in the liver^11^. Mechanistically, we previously demonstrated that TBK1 regulates ACSL1 localization to mitochondria, supporting fatty acid channeling into β-oxidation. Thus, whereas adipocyte TBK1 primarily governs whole-body energy expenditure via AMPK regulation, hepatic TBK1 acts locally to maintain mitochondrial fatty acid utilization and mitochondrial quality control. This tissue specificity highlights the versatile regulatory capacity of TBK1 across metabolic tissues and underscores the importance of understanding context-dependent TBK1 signaling.

We note that numerous studies have demonstrated that treatment with the TBK1/IKKε inhibitor amlexanox ameliorates hepatic steatosis, inflammation and fibrosis in MASLD models^36–40^. While improvements in body weight, glucose homeostasis and fat mass have been attributed to TBK1/IKKε inhibition^38,40^, these effects are mediated through multiple organ systems, including adipose tissue, brain and liver. Moreover, amlexanox exhibits pleiotropic actions beyond TBK1 and IKKε, including modulation of GRK5^41,42^, HSP90^43^, S100A13^44^ and FGF1^45^ signaling pathways, although these effects occur at higher doses. A primary effect of amlexanox is to increase energy expenditure in adipocytes via improvement in catecholamine sensitivity, including adipocyte FGF21 and IL-6 induction, which improve metabolic outcomes, suppress hepatic gluconeogenesis and promote glycemic control^38^. These data support the idea that the beneficial effects of amlexanox in MASLD may result primarily from systemic metabolic improvements, rather than direct inhibition of hepatocyte TBK1. Furthermore, amlexanox reduces inflammatory responses in various models^36,37,46–48^. Indeed, amlexanox exhibits cell-type-specific effects on immune regulation: it has been reported to promote T cell activation while suppressing macrophage-mediated pro-inflammatory responses^49^. Moreover, amlexanox enhances bile acid synthesis and promotes fecal bile acid secretion in the gut^39^, markedly improved MASH-related dyslipidemia, hepatic steatosis, inflammation, liver injury and hepatic fibrosis. In addition, amlexanox has shown clear direct effects in the liver, improving dyslipidemia and preventing atherosclerosis^50^. Thus, irrespective of the mechanism, the profound antisteatotic effects of amlexanox seen in mouse models of MAFLD and MASH are likely to prevent subsequent mitochondrial dysfunction, thus obscuring the potential effects of TBK1 inhibition on mitochondrial quality control.

Collectively, our findings support a model in which TBK1 acts as a metabolic rheostat, integrating inflammatory and nutrient-derived signals to orchestrate mitochondrial quality control and hepatic lipid metabolism, particularly important in the later stages of liver disease. Loss of TBK1 function disrupts this regulatory network, contributing to the accumulation of dysfunctional mitochondria, hepatocellular stress, and fibrosis—hallmarks of MASH pathogenesis. Restoration of TBK1 expression in mouse models improved mitophagy, reduced fibrosis-related gene expression and ameliorated liver pathology.

## Supplementary information

Supplementary Information
