# Supplementary material for: TANK-binding kinase 1 protects against MASH progression via mitochondrial quality control
Source: Exp Mol Med. 2026 Mar 13;58(3):917–31. doi: 10.1038/s12276-026-01672-9 (PMC13049026; doi:10.1038/s12276-026-01672-9)
Supplement: Supplementary file 1 — Supplementary Information [file 12276_2026_1672_MOESM1_ESM.pdf]

Supplementary Fig. 1

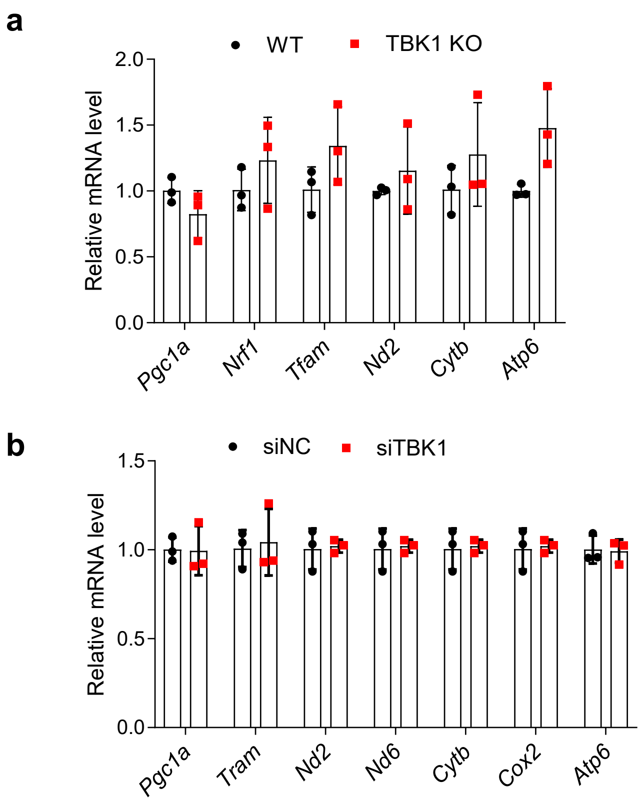

Supplementary Fig. 1 Mitochondrial biogenesis-related genes are not affected by TBK1 deficiency in HepG2 cells (a) or in primary hepatocytes (b). Data are presented as mean ± SD. Statistical significance was determined by unpaired two-tailed Student's t-test .

Supplementary Fig. 2

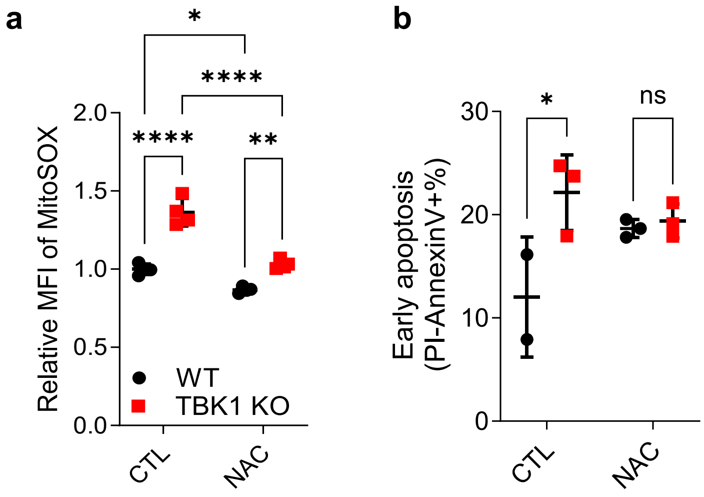

Supplementary Fig. 2 ROS scavenger rescues cell death in TBK1-deficient HepG2 cells. (a) An increased mitochondrial ROS in TBK1-deficient HepG2 cells is reduced by ROS scavenger N-acetylcysteine (NAC) treatment. (b) NAC reduces elevated cell death in TBK1-deficient cells upon NAC treatment. *$p < 0.05$, **$p < 0.01$, ****$p < 0.0001$. Data are presented as mean ± SD. Statistical significance was determined by two-way ANOVA (a-b).

Supplementary Fig. 3

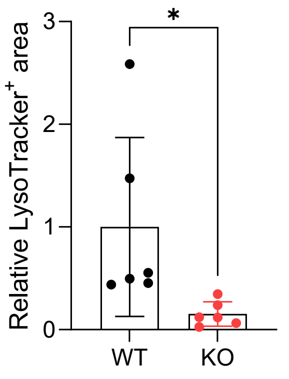

Supplementary Fig. 3 Lysotracker signal is decreased in TBK1 KO HepG2 cells. *p < 0.05. Data are presented as mean ± SD. Statistical significance was determined by unpaired two-tailed Student’s t-test .

Supplementary Fig. 4

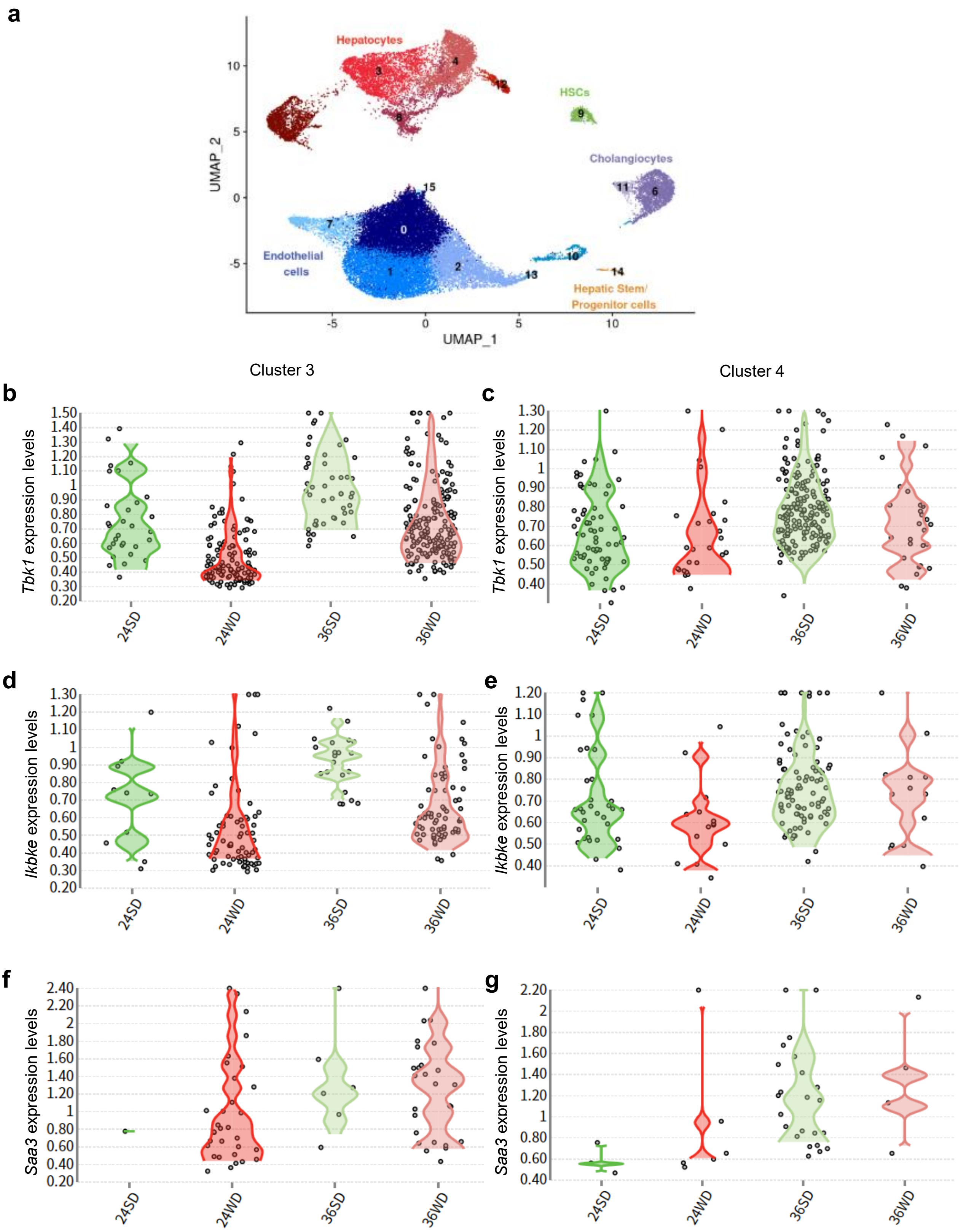

Supplementary Fig. 4 TBK1 mRNA expression level in hepatocytes by using single cell RNAseq data (Liver Cell Atlas). The mice were fed standard diet (SD) or western diet (WD) for 24 weeks or 36 weeks. (a) single cell UMAP plot in this data sets. TBK1 mRNA level in two kinds of hepatocyte clusters, 3 (b) and 4 (c). *Ikbke* mRNA level in cluster 3 (d) and cluster 4 (e). *Saa3* expression levels in cluster 3 (f) and 4 (g).

Supplementary Fig. 5

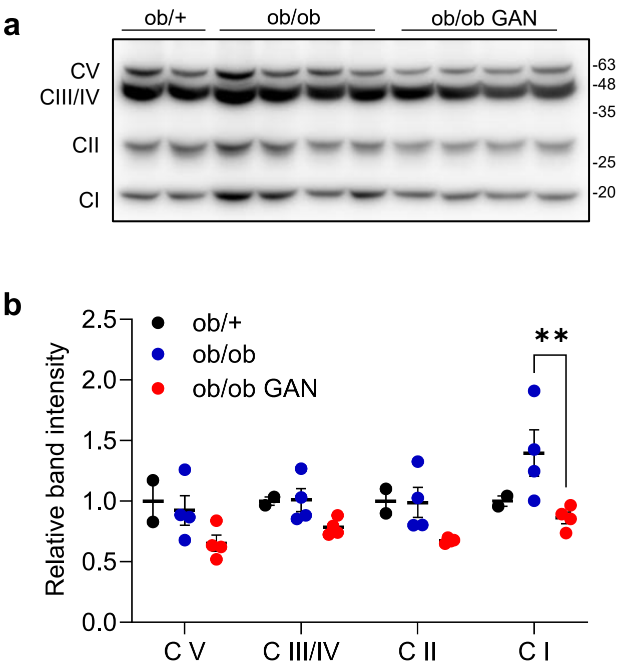

Supplementary Fig. 5 Mitochondrial OXPHOS complex are reduced in the GAN diet–induced *ob/ob* MASH model. (a) Immunoblot analysis of OXPHOS complex proteins in liver tissues from NCD-fed ob/+ (lean), NCD-fed *ob/ob* (steatosis), and GAN diet-fed *ob/ob* (MASH) mice. (b) Quantification of each complex protein intensity. **p < 0.01. Data are presented as mean ± SEM. Statistical significance was determined by t-test between *ob/ob* vs. *ob/ob* GAN.

Supplementary Fig. 6

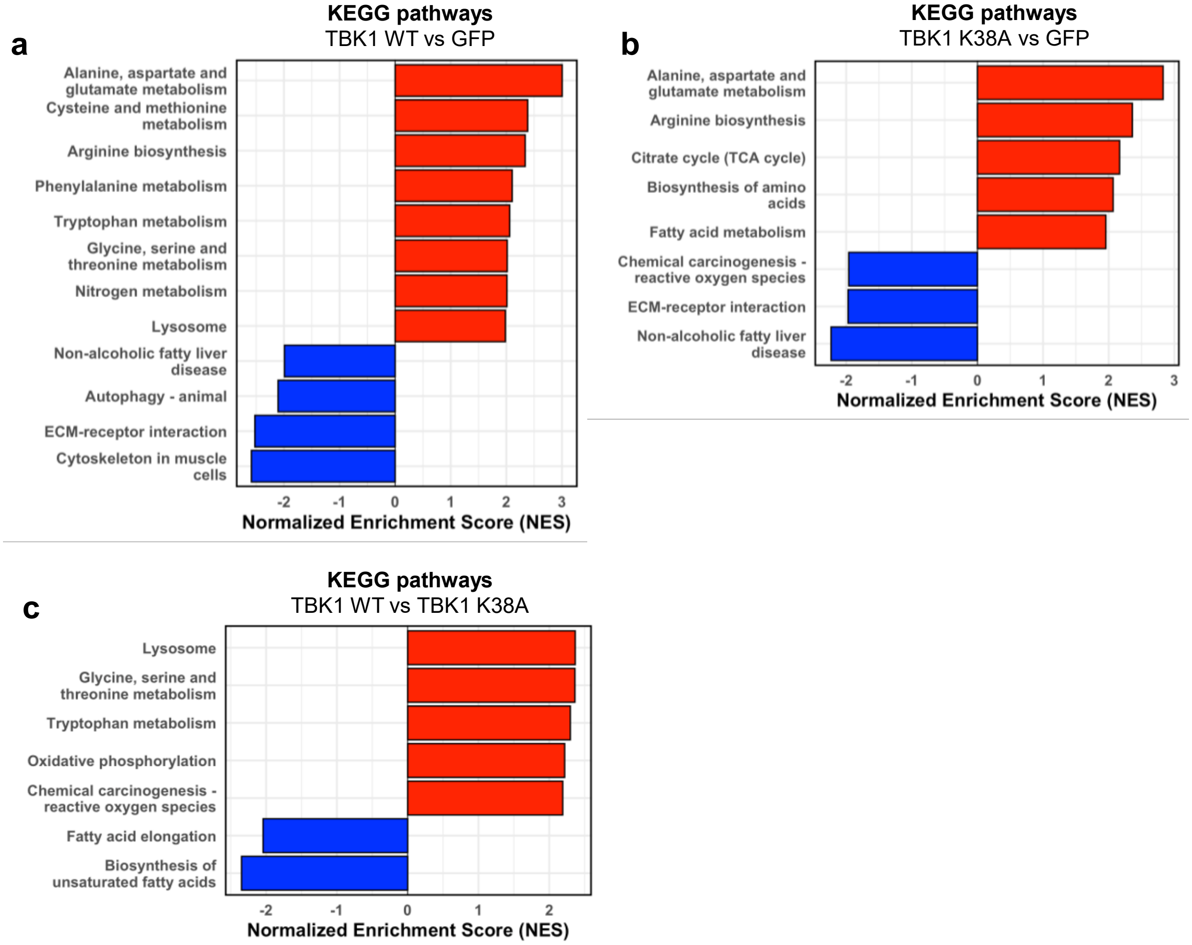

Supplementary Fig. 6 KEGG pathway enrichment analysis of RNA-seq data from liver tissues of GFP, AAV8-TBK1 WT, and AAV-TBK1 $K^{38}A$ groups. (a) A bar plot showing the normalized enrichment scores (NES) for the top pathways in the comparison between GFP and TBK1 WT. While ECM-receptor interaction and other pathways involved in liver disease and fibrosis were significantly downregulated (negative NES, blue), TBK1 WT showed significant enrichment of lysosome-related pathways (positive NES, red). (b) A similar but weaker pattern was observed between GFP and TBK1 K38A, suggesting that the kinase-dead mutant exhibited limited regulatory activity. (c) TBK1 WT versus TBK1 K38A demonstrated higher enrichment of oxidative phosphorylation and lysosomal pathways, highlighting the significance of TBK1 kinase activity for maintaining mitochondrial and lysosomal homeostasis.

Supplementary Fig. 7

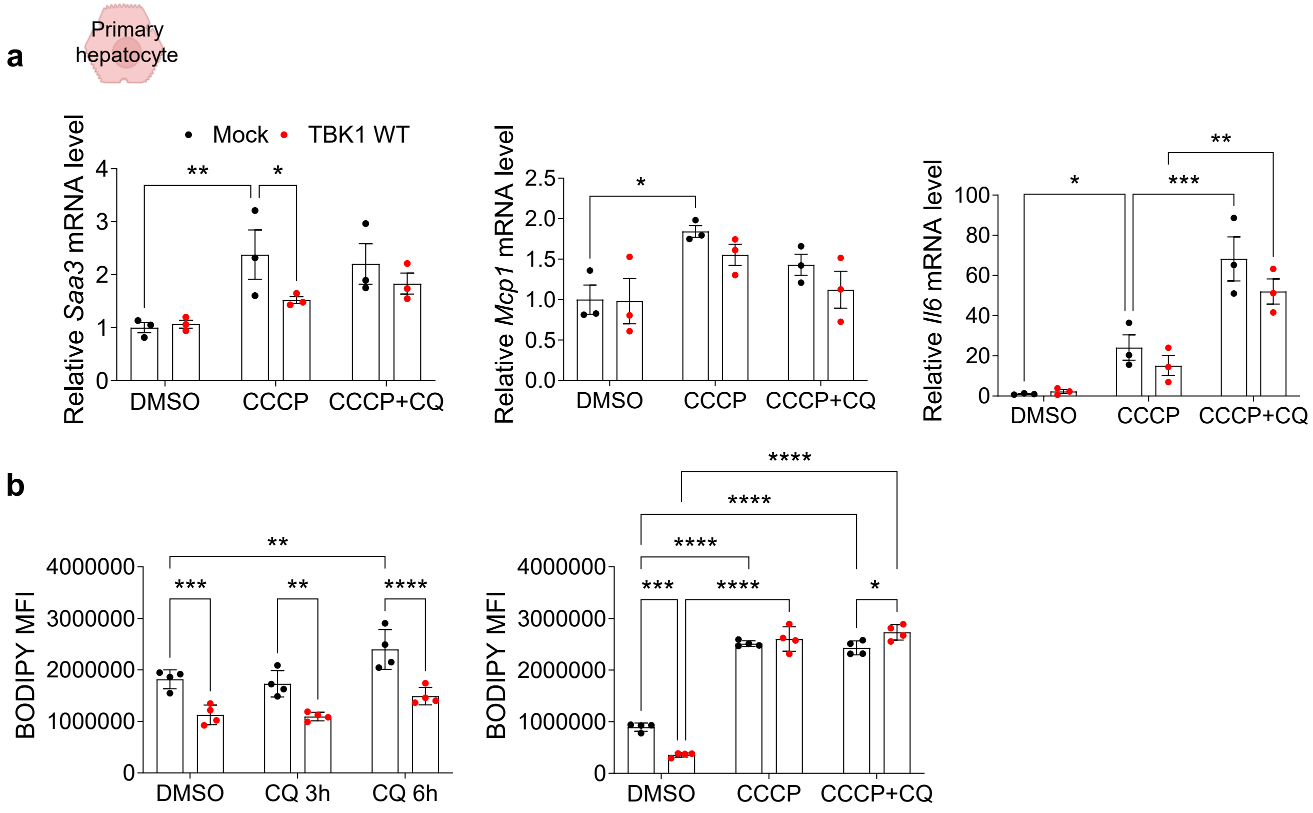

Supplementary Fig. 7 TBK1 overexpression modulates inflammatory gene expression and lipid accumulation in primary hepatocytes. (a) mRNA expression of inflammatory markers (*Saa3, Mcp1, and Il6*) in TBK1-overexpressing primary hepatocytes upon CCCP (10 μM) treatment or CCCP and CQ (30 μM) co-treatment for 6 h. TBK1 partially reduced CCCP-induced inflammatory gene expression, whereas CQ co-treatment diminished this effect, indicating a protective effect against impaired mitophagy-mediated inflammatory response. (b) Lipid accumulation was measured by BODIPY staining under basal, CQ-treated, CCCP-treated, or CCCP+CQ conditions in primary hepatocytes overexpressing TBK1. Overexpressing TBK1 decreases lipid accumulation in both basal and CQ-treated cells, while CCCP-induced lipid accumulation was not rescued. *$p < 0.05$, **$p < 0.01$, ***$p < 0.001$, ****$p < 0.0001$. Data are presented as mean ± SD. Statistical significance was determined by two-way ANOVA (a-b).

Supplementary Fig. 8

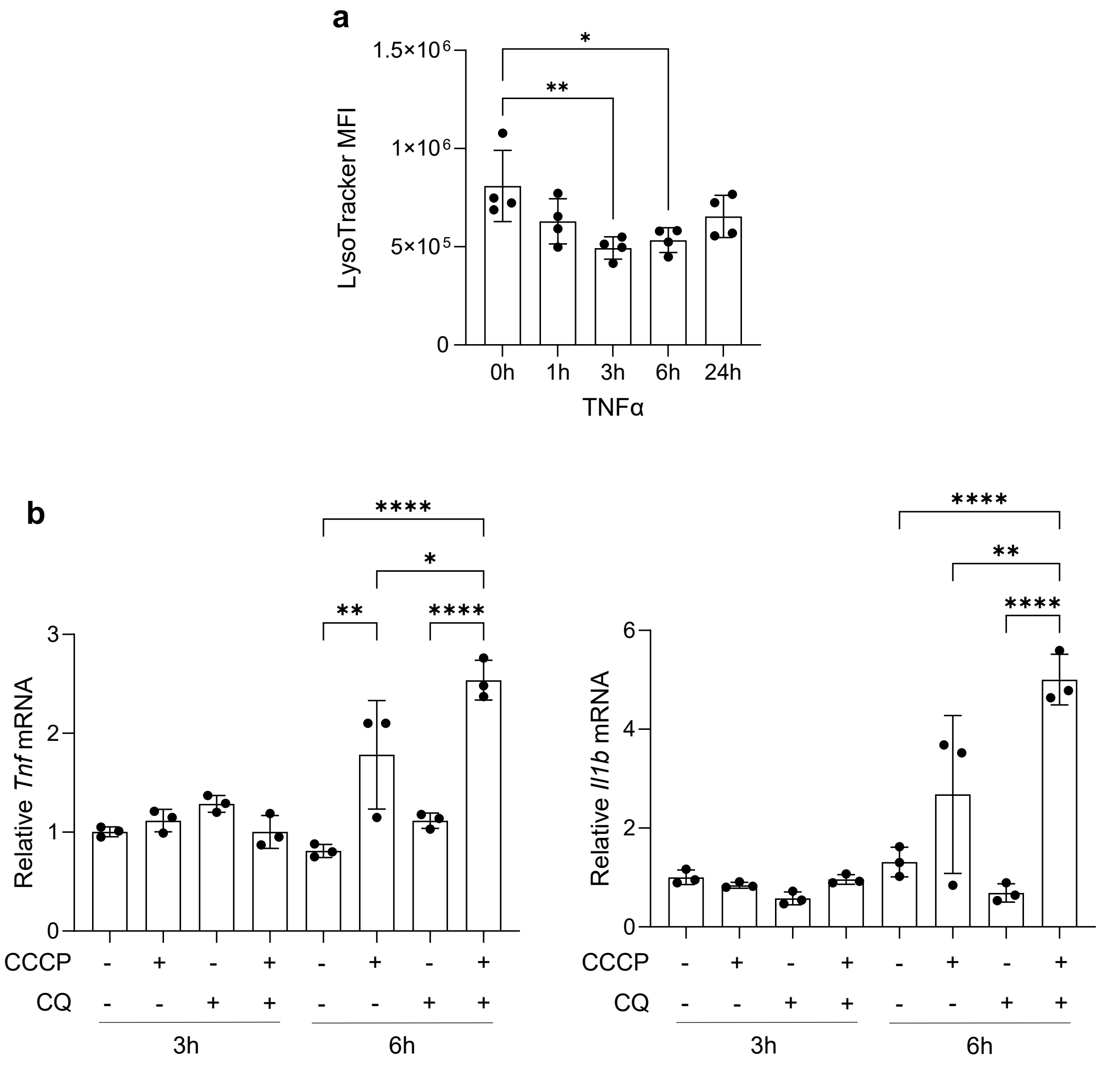

Supplementary Fig. 8 TNFα can induce lysosomal dysfunction (a) and reduced lysosome activity can also induce inflammation (b) in mouse primary hepatocytes. *$p < 0.05$, **$p < 0.01$, ****$p < 0.0001$. Data are presented as mean ± SD. Statistical significance was determined by two-way ANOVA (a-b).

Supplementary Fig. 9

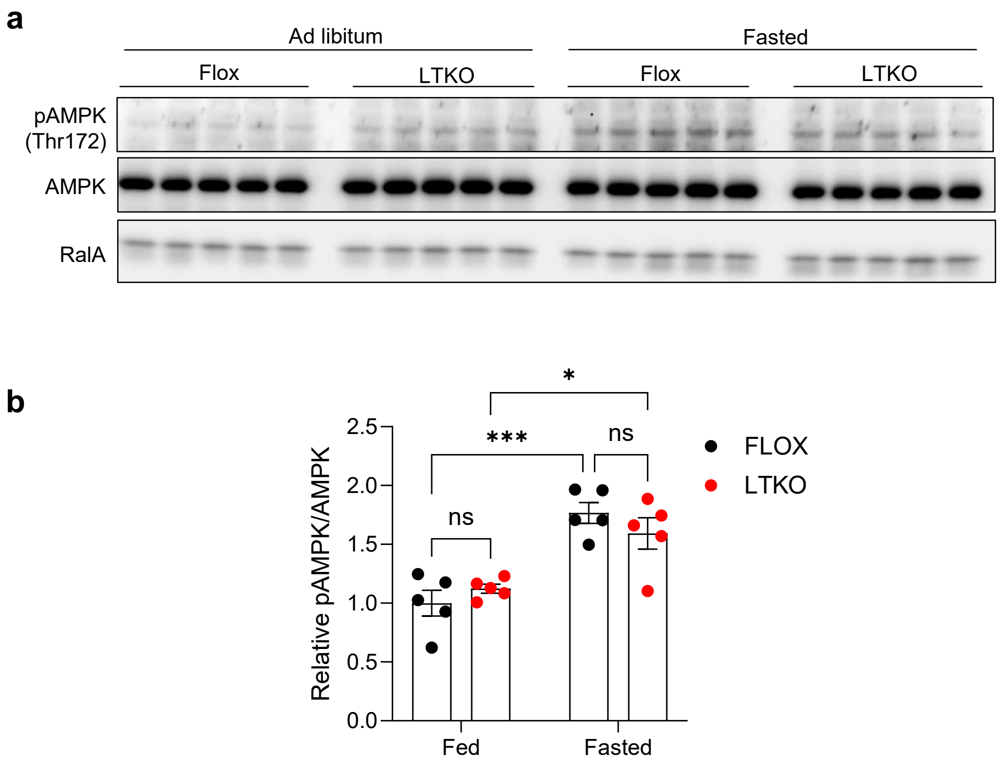

Supplementary Fig. 9 AMPK activity in LTKO mouse. Liver lysates from NCD-fed Flox and LTKO mice in fed and 16 h fasted condition were analyzed to assess AMPK activity. (a) Immunoblot analysis of phosphorylated AMPK (pAMPK) and total AMPK. (b) Quantification of the pAMPK/total AMPK ratio. NS, not significant, $*p < 0.05$, $***p < 0.001$. Data are presented as mean ± SEM. Statistical significance was determined by two-way ANOVA.

Supplementary Table 1

Gene	Forward (5'->3')	Reverse (5'->3')
Mouse Atp6v1d	GAGCACAGACTGGTCGAAA	AGCTGTCAGTTCCTTCGTGG
Mouse Atp6v1e1	ATACCACGGCCTTACTGTGC	CAGAGGATTGAGCTGTGCCA
Mouse Atp6v1h	ATGAGTACCGGTTTGCCTGG	GACTGAATGCCAGGAGCCAT
Mouse Bax	TGGAGATGAACTGGACAGCA	GATCAGCTCGGGCACTTTAG
Mouse Bcl2	AGGAGCAGGTGCCTACAAGA	GCATTTTCCCACCACTGTCT
Mouse Cd11b	ATGGACGCTGATGGCAATACC	TCCCCATTCACGTCTCCCA
Mouse Cd11c	CAAGAAGCACCGAACATGGTT	GTCTGAGCTAGAGTCACTGGT
Mouse Col1a1	GTGCTCCTGGTATTGCTGGT	GGCTCCTCGTTTTCCTTCTT
Mouse Col3a1	GGGTTTCCCTGGTCCTAAAG	CCTGGTTTCCCATTTTCTCC
Mouse Col6a1	GATGAGGGTGAAGTGGGAGA	CAGCACGAAGAGGATGTCAA
Mouse Ctsc	TGCCACATCTGAGGAACAAA	CACCAGGACTCCTCTGCATT
Mouse Ctsa	CAGCCCTCTTTCCGGCAATA	TTTGGGTCGTTCTGCGACTC
Mouse Ctsd	TACTCCATGCAGTCATCGCC	GACGACTGTGAAACACTGCG
Mouse Cyclophilin	GTG GTC TTT GGG AAG GTG AA	TTA CAG GAC ATT GCG AGC AG
Mouse Il1b	GCAACTGTTCCTGAACTCAACT	ATCTTTTGGGGTCCGTCAACT
Mouse Il6	TAGTCCTTCCTACCCCAATTTCC	TTGGTCCTTAGCCACTCCTTC
Mouse Lamp1	AGCATACCGGTGTGTCAGTG	GTTGGGGAAGGTCCATCCTG
Mouse Mcoln1	CTGACCCCCAATCCTGGGTAT	GGCCCGGAACTTGTCACAT
Mouse Mcp1	AGGTCCCTGTCATGCTTCTTG	TCTGGACCCATTCCTTCTTG
Mouse Saa3	AGCGATGCCAGAGAGGCTGT	ACCCAGTAGTTGCCCCTCTT
Mouse Tbk1	ACTGGTGATCTCTATGCTGTCA	TTCTGGAAGTCCATACGCATTG
Human Tbk1	CGGAGACCCGGCTGGTATAA	ATCCACTGGACGAAGGAAGC
Mouse Tbp	GGGAGAATCATGGACCAGAA	CCGTAAGGCATCATTGGACT
Mouse Tfeb	CCAGAAGCGAGAGCTCACAGAT	TGTGATTGTCTTTCTTCTGCCG

Supplementary Table 1. Primer sequences for qRT-PCR

Supplementary Table 2

Gene	Forward (5'->3')	Reverse (5'->3')
Human mtDNA	GCCTTCCCCCGTAAATGATA	TTATGCGATTACCGGGCTCT
Human ATPase6	AACGAAAATCTGTTCGCTTCAT	ATGTGTTGTCGTGCAGGTAGAG
Human tRNA	CACCCAAGAACAGGGTTTGT	TGGCCATGGGTATGTTGTTA
Human β2m	TGCTGTCTCCATGTTTGATGTATCT	TCTCTGCTCCCCACCTCTAAGT
Mouse mtDNA	CCCAGCTACTACCATCATTCAAGT	GATGGTTTGGGAGATTGGTTGATGT
Mouse 16s	CCGCAAGGGAAAGATGAAAGAC	TCGTTTGGTTTCGGGGTTTC
Mouse ND1	CTAGCAGAAACAAACCGGGC	CCGGCTGCGTATTCTACGTT
Mouse HK2	GCCAGCCTCTCCTGATTTTAGTGT	GGGAACACAAAAGACCTCTTCTGG

Supplementary Table 2. Primer sequences for mitochondrial DNA quantification.
